# The 5th edition of the World Health Organization Classification of Haematolymphoid Tumours: Lymphoid Neoplasms

**DOI:** 10.1038/s41375-022-01620-2

**Published:** 2022-06-22

**Authors:** Rita Alaggio, Catalina Amador, Ioannis Anagnostopoulos, Ayoma D. Attygalle, Iguaracyra Barreto de Oliveira Araujo, Emilio Berti, Govind Bhagat, Anita Maria Borges, Daniel Boyer, Mariarita Calaminici, Amy Chadburn, John K. C. Chan, Wah Cheuk, Wee-Joo Chng, John K. Choi, Shih-Sung Chuang, Sarah E. Coupland, Magdalena Czader, Sandeep S. Dave, Daphne de Jong, Ming-Qing Du, Kojo S. Elenitoba-Johnson, Judith Ferry, Julia Geyer, Dita Gratzinger, Joan Guitart, Sumeet Gujral, Marian Harris, Christine J. Harrison, Sylvia Hartmann, Andreas Hochhaus, Patty M. Jansen, Kennosuke Karube, Werner Kempf, Joseph Khoury, Hiroshi Kimura, Wolfram Klapper, Alexandra E. Kovach, Shaji Kumar, Alexander J. Lazar, Stefano Lazzi, Lorenzo Leoncini, Nelson Leung, Vasiliki Leventaki, Xiao-Qiu Li, Megan S. Lim, Wei-Ping Liu, Abner Louissaint, Andrea Marcogliese, L. Jeffrey Medeiros, Michael Michal, Roberto N. Miranda, Christina Mitteldorf, Santiago Montes-Moreno, William Morice, Valentina Nardi, Kikkeri N. Naresh, Yasodha Natkunam, Siok-Bian Ng, Ilske Oschlies, German Ott, Marie Parrens, Melissa Pulitzer, S. Vincent Rajkumar, Andrew C. Rawstron, Karen Rech, Andreas Rosenwald, Jonathan Said, Clémentine Sarkozy, Shahin Sayed, Caner Saygin, Anna Schuh, William Sewell, Reiner Siebert, Aliyah R. Sohani, Reuben Tooze, Alexandra Traverse-Glehen, Francisco Vega, Beatrice Vergier, Ashutosh D. Wechalekar, Brent Wood, Luc Xerri, Wenbin Xiao

**Affiliations:** 1 Pathology Unit, Department of Laboratories, Bambino Gesu Children’s Hospital, IRCCS, Rome, Italy; 2 Department of Pathology, University of Miami, Miami, FL USA; 3 Institute of Pathology, Julius-Maximilians-Universität Würzburg, Würzburg, Germany; 4 Department of Histopathology, Royal Marsden Hospital, London, UK; 5 Department of Pathology, Federal University of Bahia (UFBA), Salvador, Brazil; 6 University of Milan, Fondazione Cà Granda, IRCCS, Ospedale Maggiore Policlinico, Milan, Italy; 7 Department of Pathology and Cell Biology, Columbia University Irving Medical Center, New York, NY USA; 8 Division of Histopathology, SL Raheja Hospital, Mumbai, India; 9 Department of Pathology, University of Michigan, Ann Arbor, MI USA; 10 Centre for Haemato-Oncology, Barts Cancer institute, QMUL and SIHMDS Barts Health NHS Trust, London, UK; 11 Department of Pathology and Laboratory Medicine, Weill Cornell Medicine, New York, NY USA; 12 Department of Pathology, Queen Elizabeth Hospital, Kowloon, Hong Kong; 13 National University Cancer Institute, Singapore, Singapore; 14 Department of Pathology, The University of Alabama at Birmingham, Birmingham, AL USA; 15 Department of Pathology, Chi-Mei Medical Center, Tainan, Taiwan; 16 Liverpool Clinical Laboratories, Liverpool University Hospitals Foundation Trust, Liverpool, UK; 17 Department of Pathology and Laboratory Medicine, Indiana University, Indianapolis, IN USA; 18 Center for Genomic and Computational Biology and Department of Medicine, Duke University, Durham, NC USA; 19 Amsterdam UMC, location Vrije Universiteit Amsterdam, Department of Pathology, Amsterdam, The Netherlands; 20 Division of Cellular and Molecular Pathology, Department of Pathology, University of Cambridge, Cambridge, UK; 21 Department of Pathology and Laboratory Medicine, University of Pennsylvania, Philadelphia, PA USA; 22 Department of Pathology, Massachusetts General Hospital and Harvard Medical School, Boston, MA USA; 23 Department of Pathology, Stanford University School of Medicine, Stanford, CA USA; 24 Department. of Dermatology, Northwestern University Feinberg Medical School, Chicago, IL USA; 25 Department of Pathology, Tata Memorial Hospital, Mumbai, India; 26 Department of Pathology, Boston Children’s Hospital, Boston, MA USA; 27 Translational and Clinical Research Institute, Newcastle University Centre for Cancer, Faculty of Medical Sciences, Newcastle University, Newcastle-upon-Tyne, UK; 28 Dr. Senckenberg Institute of Pathology, Goethe University Frankfurt, Frankfurt am Main, Germany; 29 Hematology/Oncology, Universitätsklinikum Jena, Jena, Germany; 30 Leiden University Medical Center, Department of Pathology, Leiden, The Netherlands; 31 Department of Pathology and Laboratory Medicine, Nagoya, Japan; 32 Kempf und Pfaltz Histologische Diagnostik Zurich, and Department of Dermatology, University Hospital Zurich, Zurich, Switzerland; 33 Department of Hematopathology, Division of Pathology and Laboratory Medicine, The University of Texas MD Anderson Cancer Center, Houston, TX USA; 34 Department of Virology, Nagoya University Graduate School of Medicine, Nagoya, Japan; 35 Department of Pathology, Hematopathology Section and Lymph Node Registry, University Hospital Schleswig-Holstein, University of Kiel, Kiel, Germany; 36 Department of Pathology and Laboratory Medicine, Children’s Hospital Los Angeles, Los Angeles, CA USA; 37 Division of Hematology, Mayo Clinic, Rochester, MN USA; 38 Departments of Pathology & Genomic Medicine, The University of Texas MD Anderson Cancer Center, Houston, TX USA; 39 Department of Medical Biotechnology, University of Siena, Siena, Italy; 40 Division of Nephrology and Hypertension, Division of Hematology, Mayo Clinic, Rochester, MN USA; 41 Department of Pathology, Medical College of Wisconsin and Children’s Wisconsin, Milwaukee, WI USA; 42 Department of Pathology, Fudan University Shanghai Cancer Center, Shanghai, China; 43 Department of Pathology, West-China Hospital, Sichuan University, Chengdu, China; 44 Department of Pathology & Immunology, Baylor College of Medicine and Texas Children’s Hospital, Houston, TX USA; 45 Department of Pathology, Charles University in Prague, Faculty of Medicine in Plzen, Plzen, Czech Republic; 46 Department of Dermatology, Venereology and Allergology, University Medical Center Göttingen, Göttingen, Germany; 47 Anatomic Pathology Department and Translational Hematopathology Lab, Valdecilla/IDIVAL University Hospital, Santander, Spain; 48 Department of Laboratory Medicine and Pathology, Mayo Clinic, Rochester, MN USA; 49 Section of Pathology, Clinical Research Division, Fred Hutchinson Cancer Center, Seattle, WA USA; 50 Department of Pathology, Yong Loo Lin School of Medicine, National University of Singapore, Singapore, Singapore; 51 Department of Clinical Pathology, Robert-Bosch-Krankenhaus, and Dr. Margarete Fischer-Bosch Institute of Clinical Pharmacology, Stuttgart, Germany; 52 Department of Pathology, Bordeaux University Hospital, Bordeaux, France; 53 Department of Pathology and Laboratory Medicine, Memorial Sloan Kettering Cancer Center, New York, NY USA; 54 Division of Hematology, Mayo Clinic, Rochester, Minnesota, Rochester, MN USA; 55 HMDS, Leeds Cancer Centre, Leeds Teaching Hospitals NHS Trust, Leeds, UK; 56 Department of Pathology and Laboratory Medicine, University of California Los Angeles, Los Angeles, CA USA; 57 MD-PhD, DITEP, Gustave Roussy, Villejuif, France; 58 Department of Pathology- Aga Khan University Hospital- Nairobi, Nairobi, Kenya; 59 Section of Hematology/Oncology, University of Chicago, Chicago, IL USA; 60 Department of Oncology, University of Oxford, Oxford, UK; 61 Immunology Division, Garvan Institute of Medical Research, Sydney, Australia; 62 Institute of Human Genetics, Ulm University and Ulm University Medical Center, Ulm, Germany; 63 Division of Haematology and Immunology, Leeds Institute of Medical Research, University of Leeds, Leeds, UK; 64 Hospices Civils de Lyon/Department of Pathology/ Université Lyon 1/ Centre International de Recherche en Infectiologie (CIRI) INSERM U1111 -, CNRS UMR5308 Lyon, France; 65 Department of Pathology, Hopital Haut-Lévêque, CHU Bordeaux, Pessac, France; 66 National Amyloidosis Centre, University College London, London, UK; 67 Department of Pathology, Institut Paoli-Calmettes and Aix-Marseillreference University, Marseille, France

**Keywords:** Diagnosis, Lymphoma

## Abstract

We herein present an overview of the upcoming 5^th^ edition of the World Health Organization Classification of Haematolymphoid Tumours focussing on lymphoid neoplasms. Myeloid and histiocytic neoplasms will be presented in a separate accompanying article. Besides listing the entities of the classification, we highlight and explain changes from the revised 4^th^ edition. These include reorganization of entities by a hierarchical system as is adopted throughout the 5^th^ edition of the WHO classification of tumours of all organ systems, modification of nomenclature for some entities, revision of diagnostic criteria or subtypes, deletion of certain entities, and introduction of new entities, as well as inclusion of tumour-like lesions, mesenchymal lesions specific to lymph node and spleen, and germline predisposition syndromes associated with the lymphoid neoplasms.

## Introduction

Evidence-based classification of disease is fundamental for the treatment of individual patients, monitoring of global disease incidence, and investigating all aspects of disease causation, prevention and therapy. The World Health Organization (WHO) classification of lymphoid tumours has provided a global reference for the diagnosis of lymphoid neoplasms since its 3^rd^ edition in 2001 [1] which was based on the R.E.A.L Classification developed by the International Lymphoma Study Group (ILSG) in the early 1990s [2]. The definitions laid down in the successive WHO classifications [3, 4] have not only been adopted for use by pathologists, clinicians, and basic and translational research scientists, but they have also been incorporated into the International Classification of Diseases (ICD) codes, and thereby serve as a global reference for epidemiological monitoring across national and international health policy organizations. In this article, we provide the conceptual framework and major developments in lymphoid neoplasms in the upcoming 5^th^ edition of the WHO Classification of Haematolymphoid Tumours (WHO-HAEM5) scheduled to be published in 2022. An overview of myeloid neoplasms will be published separately.

The International Agency for Research on Cancer (IARC) initiated the process culminating in WHO-HAEM5 in 2018 by laying out the governance rules and classification principles for the entire 5^th^ Edition series of the WHO classification of tumours, comprising 14 volumes, each dedicated to neoplasia of specific organ systems and/or clinical contexts (Paediatric Tumours and Genetic Tumour Syndromes). In 2021, expert members of the editorial board and authors were invited to contribute to WHO-HAEM5 based on their records of diagnostic and/or scientific expertise, regional representation, equity and lack of potential conflicts-of-interest. For most chapters, a multidisciplinary author team was formed including haematopathologists, haematologists, oncologists, geneticists, epidemiologists and molecular biologists. Experts from other disciplines, such as radiation oncologists and immunologists, were also involved where appropriate. Author teams worked “virtually”, in close collaboration, further supported by regular online meetings with the editorial team despite (and possibly in part thanks to) the challenges encountered during the COVID-19 pandemic. In addition, major issues arising during the development of the classification were discussed, resolved and harmonized across entities, both within WHO-HAEM5 and across other WHO volumes that cover some of the same entities in different clinical and/or organ-specific contexts. This was accomplished via regular meetings among expert groups and further dedicated conferences, including a clinical forum with all clinicians involved in the WHO-HAEM5. Public consultation was sought on an initial classification draft. Final decisions were taken based on principles of evidence-based medicine.

The resulting WHO-HAEM5 is a systematic evolution of the prior classifications. To allow for continuity in daily practice and ongoing clinical trials, a relatively conservative approach was taken in making changes to nomenclature. The WHO-HAEM5, like all 5th Edition WHO tumour volumes, applies a hierarchical system for classification. That is, it organises diseases in order of increasing levels of specification: category (e.g., mature B-cell), family/class (e.g., large B-cell lymphomas), entity/type (e.g., diffuse large B-cell lymphoma, not otherwise specified) and subtype (e.g., diffuse large B cell lymphoma, not otherwise specified, germinal center B-cell-like). Entities and subtypes have been formulated such that the implementation of the WHO-HAEM5 classification system is possible globally, in all settings. The WHO-HAEM5 recognizes the increasing importance of genetic and other molecular data in the evaluation of lymphoid neoplasia; however, consideration has also been given to the fact that the required diagnostic resources are not universally available. Thus, to facilitate a pragmatic approach to diagnosis while also encouraging the adoption of molecular testing where required, “essential“ and “desirable“ diagnostic criteria for each entity are defined in a hierarchical way. “Essential criteria” are minimal criteria to allow the diagnosis of an entity as universally as possible, although molecular criteria are inevitably included for some entities. “Desirable criteria” are those that aid in confirmation and refinement of the diagnosis, and usually require the application of advanced techniques. In circumstances where resources are not available to reach a definitive diagnosis of an entity (or when suboptimal quality or quantity of material is limiting), a diagnostic label based on the family name of that entity can be applied.

Provisional entities were not created in WHO-HAEM5 as these, by definition, lack sufficient evidence. Novel potential subtypes have been restrictively proposed for some entities, such as in Burkitt lymphoma, where besides the three traditional epidemiologic variants, the distinction of EBV-positive and EBV-negative Burkitt lymphoma subtypes is recommended.

The order of classification follows the traditional major subgrouping according to cell lineage, with precursor cell neoplasms followed by mature malignancies. Within a family, the entities are generally arranged in an order commencing with more indolent and progressing to increasingly aggressive ones. For the first time, in an effort to prevent the over-diagnosis of lymphoma and to improve the recognition of clinicopathologically distinct entities, non-neoplastic conditions mimicking lymphoma or representing an important differential diagnosis, have been included in WHO-HAEM5. Similarly, in light of the increasing clinical importance of germline tumour predisposition syndromes, which are frequently associated with lymphoid neoplasms, such as ataxia telangiectasia, dedicated chapters have been introduced. In addition, the rapid development in the understanding of lymphoid proliferations associated with inborn errors of immunity (primary immunodeficiencies) and acquired immune disorders justified significant updates, and these have been included in WHO-HAEM5.

The following sections represent an overview of the most significant changes made in WHO-HAEM5 compared with WHO-HAEM4R (Tables 1–3).

**Table 1:** WHO Classification of Haematolymphoid Tumours, 5^th^ edition: B-cell lymphoid proliferations and lymphomas.

WHO Classification, 5^th^ edition	WHO Classification, revised 4^th^ edition
***Tumour-like lesions with B-cell predominance***	
Reactive B-cell-rich lymphoid proliferations that can mimic lymphoma	*Not previously included*
IgG4-related disease	*Not previously included*
Unicentric Castleman disease	*Not previously included*
Idiopathic multicentric Castleman disease	*Not previously included*
KSHV/HHV8-associated multicentric Castleman disease	Multicentric Castleman disease
**Precursor B-cell neoplasms**	
***B-cell lymphoblastic leukaemias/lymphomas***	
B-lymphoblastic leukaemia/lymphoma, NOS	(Same)
B-lymphoblastic leukaemia/lymphoma with high hyperdiploidy	B-lymphoblastic leukaemia/lymphoma with hyperdiploidy
B-lymphoblastic leukaemia/lymphoma with hypodiploidy	(Same)
B-lymphoblastic leukaemia/lymphoma with iAMP21	(Same)
B-lymphoblastic leukaemia/lymphoma with *BCR*::*ABL1* fusion	B-lymphoblastic leukaemia/lymphoma with t(9;22)(q34;q11.2); *BCR-ABL1*
B-lymphoblastic leukaemia/lymphoma with *BCR*::*ABL1*-like features	B-lymphoblastic leukaemia/lymphoma, *BCR-ABL1*-like
B-lymphoblastic leukaemia/lymphoma with *KMT2A* rearrangement	B-lymphoblastic leukaemia/lymphoma with t(v;11q23.3); *KMT2A-*rearranged
B-lymphoblastic leukaemia/lymphoma with *ETV6*::*RUNX1* fusion	B-lymphoblastic leukaemia/lymphoma with t(12;21)(p13.2;q22.1); *ETV6-RUNX1*
B-lymphoblastic leukaemia/lymphoma with *ETV6*::*RUNX1*-like features	*Not previously included*
B-lymphoblastic leukaemia/lymphoma with *TCF3*::*PBX1* fusion	B-lymphoblastic leukaemia/lymphoma with t(1;19)(q23;p13.3); *TCF3-PBX1*
B-lymphoblastic leukaemia/lymphoma with *IGH*::*IL3 fusion*	B-lymphoblastic leukaemia/lymphoma with t(5;14)(q31.1;q32.1); *IGH*/*IL3*
B-lymphoblastic leukaemia/lymphoma with *TCF3*::*HLF* fusion	*Not previously included*
B-lymphoblastic leukaemia/lymphoma with other defined genetic abnormalities	(Same)
**Mature B-cell neoplasms**	
***Pre-neoplastic and neoplastic small lymphocytic proliferations***	
Monoclonal B-cell lymphocytosis	(Same)
Chronic lymphocytic leukaemia/small lymphocytic lymphoma	(Same)
(Entity deleted)	B-cell prolymphocytic leukaemia
***Splenic B-cell lymphomas and leukaemias***	
Hairy cell leukaemia	(Same)
Splenic marginal zone lymphoma	(Same)
Splenic diffuse red pulp small B-cell lymphoma	(Same)
Splenic B-cell lymphoma/leukaemia with prominent nucleoli	*Not previously included* (encompassing hairy cell leukaemia variant and some cases of B-cell prolymphocytic leukaemia)
***Lymphoplasmacytic lymphoma***	
Lymphoplasmacytic lymphoma	(Same)
***Marginal zone lymphoma***	
Extranodal marginal zone lymphoma of mucosa-associated lymphoid tissue	(Same)
Primary cutaneous marginal zone lymphoma	*Not previously included (*originally included under “extranodal marginal zone lymphoma of mucosa-associated lymphoid tissue”)
Nodal marginal zone lymphoma	(Same)
Paediatric marginal zone lymphoma	(Same)
***Follicular lymphoma***	
In situ follicular B-cell neoplasm	In situ follicular neoplasia
Follicular lymphoma	(Same)
Paediatric-type follicular lymphoma	(Same)
Duodenal-type follicular lymphoma	(Same)
***Cutaneous follicle centre lymphoma***	
Primary cutaneous follicle centre lymphoma	(Same)
***Mantle cell lymphoma***	
In situ mantle cell neoplasm	In situ mantle cell neoplasia
Mantle cell lymphoma	(Same)
Leukaemic non-nodal mantle cell lymphoma	(Same)
***Transformations of indolent B-cell lymphomas***	
Transformations of indolent B-cell lymphomas	*Not previously included*
***Large B-cell lymphomas***	
Diffuse large B-cell lymphoma, NOS	(Same)
T-cell/histiocyte-rich large B-cell lymphoma	(Same)
Diffuse large B-cell lymphoma/ high grade B-cell lymphoma with* MYC* and *BCL2* rearrangements	High-grade B-cell lymphoma with *MYC* and* BCL2* and/or *BCL6* rearrangements
ALK-positive large B-cell lymphoma	(Same)
Large B-cell lymphoma with *IRF4* rearrangement	(Same)
High-grade B-cell lymphoma with 11q aberrations	Burkitt-like lymphoma with 11q aberration
Lymphomatoid granulomatosis	(Same)
EBV-positive diffuse large B-cell lymphoma	EBV-positive diffuse large B-cell lymphoma, NOS
Diffuse large B-cell lymphoma associated with chronic inflammation	(Same)
Fibrin-associated large B-cell lymphoma	*Not previously included* (Previously considered a subtype of diffuse large B-cell lymphoma associated with chronic inflammation)
Fluid overload-associated large B-cell lymphoma	*Not previously included*
Plasmablastic lymphoma	(Same)
Primary large B-cell lymphoma of immune-privileged sites	*Not previously included*, encompassing primary diffuse large B-cell lymphoma of the CNS in revised 4^th^ edition (*plus primary large B-cell lymphoma of the vitreoretina and primary large B-cell lymphoma of the testis)*
Primary cutaneous diffuse large B-cell lymphoma, leg type	(Same)
Intravascular large B-cell lymphoma	(Same)
Primary mediastinal large B-cell lymphoma	(Same)
Mediastinal grey zone lymphoma	B-cell lymphoma, unclassifiable, with features intermediate between DLBCL and classic Hodgkin lymphoma
High-grade B-cell lymphoma, NOS	(Same)
***Burkitt lymphoma***	
Burkitt lymphoma	(Same)
***KSHV/HHV8-associated B-cell lymphoid proliferations and lymphomas***	
Primary effusion lymphoma	(Same)
KSHV/HHV8-positive diffuse large B-cell lymphoma	HHV8-positive diffuse large B-cell lymphoma, NOS
KSHV/HHV8-positive germinotropic lymphoproliferative disorder	HHV8-positive germinotropic lymphoproliferative disorder
***Lymphoid proliferations and lymphomas associated with immune deficiency and dysregulation***	
Hyperplasias arising in immune deficiency/dysregulation	*Not previously included*, encompassing non-destructive post-transplant lymphoproliferative disorders, among others
Polymorphic lymphoproliferative disorders arising in immune deficiency/dysregulation	*Not previously included*, encompassing polymorphic posttransplant lymphoproliferative disorders, other iatrogenic immunodeficiency-associated lymphoproliferative disorders, among others
EBV-positive mucocutaneous ulcer	(Same)
Lymphomas arising in immune deficiency / dysregulation	*Not previously included*, encompassing monomorphic posttransplant lymphoproliferative disorders, classic Hodgkin lymphoma posttransplant lymphoproliferative disorders, lymphomas associated with HIV infection, among others
Inborn error of immunity-associated lymphoid proliferations and lymphomas	Lymphoproliferative diseases associated with primary immune disorders
***Hodgkin lymphoma***	
Classic Hodgkin lymphoma	(Same)
Nodular lymphocyte predominant Hodgkin lymphoma	(Same)
**Plasma cell neoplasms and other diseases with paraproteins**	
***Monoclonal gammopathies***	
Cold agglutinin disease	*Not previously included*
IgM monoclonal gammopathy of undetermined significance	(Same)
Non-IgM monoclonal gammopathy of undetermined significance	(Same)
Monoclonal gammopathy of renal significance	*Not previously included*
***Diseases with monoclonal immunoglobulin deposition***	
Immunoglobulin-related (AL) amyloidosis	Primary amyloidosis
Monoclonal immunoglobulin deposition disease	Light chain and heavy chain deposition disease
***Heavy chain diseases***	
Mu heavy chain disease	(Same)
Gamma heavy chain disease	(Same)
Alpha heavy chain disease	(Same)
***Plasma cell neoplasms***	
Plasmacytoma	(Same)
Plasma cell myeloma	(Same)
Plasma cell neoplasms with associated paraneoplastic syndrome -POEMS syndrome -TEMPI syndrome -AESOP syndrome	(Same) Except AESOP syndrome *not previously included*

**Table 2:** WHO Classification of Haematolymphoid Tumours, 5^th^ edition: T-cell and NK-cell lymphoid proliferations and lymphomas.

**WHO Classification, 5**^**th**^ **edition**	**WHO Classification, revised 4**^**th**^ **edition**
**Tumour-like lesions with T-cell predominance**	
Kikuchi-Fujimoto disease	*Not previously included*
Indolent T-lymphoblastic proliferation	*Not previously included*
Autoimmune lymphoproliferative syndrome	*Not previously included*
**Precursor T-cell neoplasms**	
***T-lymphoblastic leukaemia/lymphoma***	
T-lymphoblastic leukaemia / lymphoma, NOS	T-lymphoblastic leukaemia/lymphoma
Early T-precursor lymphoblastic leukaemia / lymphoma	Early T-cell precursor lymphoblastic leukaemia
(Entity deleted)	NK-lymphoblastic leukaemia/lymphoma
**Mature T-cell and NK-cell neoplasms**	
***Mature T-cell and NK-cell leukaemias***	
T-prolymphocytic leukaemia	(Same)
T-large granular lymphocytic leukaemia	T-cell large granular lymphocytic leukaemia
NK-large granular lymphocytic leukaemia	Chronic lymphoproliferative disorder of NK cells
Adult T-cell leukaemia/lymphoma	(Same)
Sezary syndrome	(Same)
Aggressive NK-cell leukaemia	(Same)
***Primary cutaneous T-cell lymphomas***	
Primary cutaneous CD4-positive small or medium T-cell lymphoproliferative disorder	(Same)
Primary cutaneous acral CD8-positive lymphoproliferative disorder	Primary cutaneous acral CD8-positive T-cell lymphoma
Mycosis fungoides	(Same)
Primary cutaneous CD30-positive T-cell lymphoproliferative disorder: Lymphomatoid papulosis	(Same)
Primary cutaneous CD30-positive T-cell lymphoproliferative disorder: Primary cutaneous anaplastic large cell lymphoma	(Same)
Subcutaneous panniculitis-like T-cell lymphoma	(Same)
Primary cutaneous gamma/delta T-cell lymphoma	(Same)
Primary cutaneous CD8-positive aggressive epidermotropic cytotoxic T-cell lymphoma	(Same)
Primary cutaneous peripheral T-cell lymphoma, NOS	*Not previously included*
***Intestinal T-cell and NK-cell lymphoid proliferations and lymphomas***	
Indolent T-cell lymphoma of the gastrointestinal tract	Indolent T-cell lymphoproliferative disorder of the gastrointestinal tract
Indolent NK-cell lymphoproliferative disorder of the gastrointestinal tract	*Not previously included*
Enteropathy-associated T-cell lymphoma	(Same)
Monomorphic epitheliotropic intestinal T-cell lymphoma	(Same)
Intestinal T-cell lymphoma, NOS	(Same)
***Hepatosplenic T-cell lymphoma***	
Hepatosplenic T-cell lymphoma	(Same)
***Anaplastic large cell lymphoma***	
ALK-positive anaplastic large cell lymphoma	Anaplastic large cell lymphoma, ALK-positive
ALK-negative anaplastic large cell lymphoma	Anaplastic large cell lymphoma, ALK-negative
Breast implant-associated anaplastic large cell lymphoma	(Same)
**Nodal T-follicular helper (TFH) cell lymphoma**	
Nodal TFH cell lymphoma, angioimmunoblastic-type	Angioimmunoblastic T-cell lymphoma
Nodal TFH cell lymphoma, follicular-type	Follicular T-cell lymphoma
Nodal TFH cell lymphoma, NOS	Nodal peripheral T-cell lymphoma with TFH phenotype
***Other peripheral T-cell lymphomas***	
Peripheral T-cell lymphoma, not otherwise specified	(Same)
***EBV-positive NK/T-cell lymphomas***	
EBV-positive nodal T- and NK-cell lymphoma	*Not previously included*
Extranodal NK/T-cell lymphoma	Extranodal NK/T-cell lymphoma, nasal-type
***EBV-positive T- and NK-cell lymphoid proliferations and lymphomas of childhood***	
Severe mosquito bite allergy	(Same)
Hydroa vacciniforme lymphoproliferative disorder	Hydroa vacciniforme-like lymphoproliferative disorder
Systemic chronic active EBV disease	Chronic active EBV infection of T- and NK-cell type, systemic form
Systemic EBV-positive T-cell lymphoma of childhood	(Same)

**Table 3:** WHO Classification of Haematolymphoid Tumours, 5^th^ edition: Stroma-derived neoplasms of lymphoid tissues.

WHO Classification, 5^th^ edition	WHO Classification, revised 4^th^ edition
**Mesenchymal dendritic cell neoplasms**	
Follicular dendritic cell sarcoma	(Same)
EBV-positive inflammatory follicular dendritic cell sarcoma	Inflammatory pseudotumour-like follicular/fibroblastic dendritic cell sarcoma
Fibroblastic reticular cell tumour	(Same)
**Myofibroblastic tumour**	
Intranodal palisaded myofibroblastoma	*Not previously included*
**Spleen-specific vascular-stromal tumours**	
***Splenic vascular-stromal tumours***	
Littoral cell angioma	*Not previously included*
Splenic hamartoma	*Not previously included*
Sclerosing angiomatoid nodular transformation of spleen	*Not previously included*

## B-cell lymphoid proliferations and lymphomas

### New addition to WHO-HAEM5: Tumour-like lesions with B-cell predominance

For the first time, the WHO ‘Blue Book’ on haematolymphoid tumours introduces tumour-like lesions, including five entities in a distinct class of **tumour-like lesions with B-cell predominance**. Castleman disease is not a single disease but rather three clinicopathologically distinct entities: unicentric Castleman disease, idiopathic multicentric Castleman disease, and KSHV/HHV8-associated multicentric Castleman disease. The diagnostic algorithm for the classification of Castleman disease requires an integrated approach, including histological, haematological, immunological, and clinical parameters [5–9]. Also included in this section is IgG4-related disease; IgG4-related lymphadenopathy has features that can overlap with Castleman disease. The fifth chapter covers other non-neoplastic B-cell predominant lymphoid proliferations involving lymph nodes and/or extranodal sites that can mimic lymphomas, including progressive transformation of germinal centers, infectious mononucleosis, florid reactive lymphoid hyperplasia/lymphoma-like lesion of the female genital tract, and systemic lupus erythematosus.

### B-lymphoblastic leukaemias/lymphomas (B-ALL): New genetically defined entities and subtypes

Following the principles of ‘essential’ and ‘desirable’ diagnostic criteria outlined above, B-lymphoblastic leukaemia/lymphoma (B-ALL) can be diagnosed at the family/class level on morphology and immunophenotype alone as B-ALL, not further classified (NFC). Most entities can be classified based on broadly-available cytogenetic testing, although molecular genetic subtyping is required for some entities based on the current state-of-the-art. B-ALL NOS, is to be reserved for cases that cannot be classified even after comprehensive testing. The majority of precursor B-cell neoplasms are classified in WHO-HAEM5 according to ploidy changes, such as hyperdiploidy and hypodiploidy, as well as chromosomal rearrangements or the presence of other genetic drivers [10]. In most cases, well-known drivers underlie B-ALL pathogenesis: iAMP21, *BCR*::*ABL1* fusion, *KMT2A* rearrangements, *ETV6*::*RUNX1* fusion, *TCF3*::*PBX1* fusion or *IGH*::*IL3* fusion. The classification based on these groups remains largely unchanged from WHO-HAEM4R; however, the nomenclature focuses on the molecular events rather than cytogenetic alterations, to allow for the application of differing techniques for their detection (Table 1). Other minor updates reflect the incorporation of additional genetic findings and refinements in the definitions of entities based on shared gene expression features. The rare B-ALL with *TCF3*::*HLF* fusion has been added to WHO-HAEM5; it is distinct from B-ALL with *TCF3*::*PBX1* fusion and is characterized by a particularly aggressive behaviour [11, 12]. B-ALL with *BCR*::*ABL1*-like features is now an entity (previously a provisional entity), by definition sharing gene expression and phenotypic features of B-ALL with *BCR*::*ABL1* fusion; it is prevalent across all age groups [13, 14] and shows significant benefit from targeted therapies [15–17]. Similarly, advances in diagnostic methodologies have allowed identification of a new entity, B-ALL with *ETV6*::*RUNX1*-like features, the description of which follows the section on B-ALL with *ETV6*::*RUNX1* fusion [18].

Recent gene expression and sequencing studies have identified a number of novel genetic drivers that appear to confer distinct clinical, phenotypic and/or prognostic features. Considering emerging, yet limited, evidence for separating them in the future as potential novel entities, these new subtypes are subsumed under “B-ALL with other defined genetic abnormalities”. These include B-ALL with *DUX4* [18, 19], *MEF2D* [20], *ZNF384* [21] or *NUTM1* [22] rearrangements, with *IG*::*MYC* fusion [23, 24], and with *PAX5*alt [25] or PAX5 p.P80R (NP_057953.1) [26] abnormalities. Intriguingly, B-ALL with *ZNF384* rearrangement, *DUX4* rearrangement or PAX5 p.P80R may show monocytic differentiation following therapy and even at diagnosis [27, 28], broadening concepts of the plasticity of leukemic lineages. This plasticity has important implications for disease management, including minimal residual disease (MRD) assessment [27].

### Mature B-cell neoplasms

The category of mature B-cell neoplasms comprises 12 families. The hierarchical structure is outlined in Table 1.

### Pre-neoplastic and neoplastic small lymphocytic proliferations: MBL and CLL/SLL remain; B-PLL is no longer recognized as an entity

This family comprises two entities: Monoclonal B-cell Lymphocytosis (MBL) and Chronic Lymphocytic Leukaemia/Small Lymphocytic Lymphoma (CLL/SLL). WHO-HAEM5 recognizes three subtypes of **monoclonal B-cell lymphocytosis (MBL)**:

- **Low-count MBL or clonal B-cell expansion**: clonal CLL/SLL-phenotype B-cell count below 0.5 x 10^9^/L with no other features diagnostic of B-lymphoproliferative disorder. The arbitrary threshold is based on the distribution of clonal B-cell counts in population studies compared to clinical cohorts [29].
- **CLL/SLL-type MBL:** monoclonal CLL/SLL-phenotype B-cell count ≥0.5 x 10^9^/L and total B-cell count less than 5 x 10^9^/L with no other features diagnostic of CLL/SLL [30]. The threshold of less than 5 x 10^9^/L is arbitrary but identifies a group with a very low likelihood of requiring treatment compared to individuals with B-cell counts between 5–10 x 10^9^/L [31].
- **non-CLL/SLL-type MBL:** ANY monoclonal non-CLL/SLL phenotype B-cell expansion with no symptoms or features diagnostic of another mature B-cell neoplasm. The majority of cases have features consistent with a marginal zone (MZ) origin [32].

All subtypes of MBL are clinically characterized by immune impairment with sub-optimal response to vaccinations and increased risk of infection [33–37]. In the diagnosis of **CLL**, CD5, CD19, CD20, CD23, and surface or cytoplasmic kappa and lambda light chains are regarded as essential markers, and CD10, CD43, CD79b, CD81, CD200 and ROR1 as additional targets useful in the differential diagnosis from other small B-cell lymphomas/leukaemias [38]. In addition to del(11q), del(13q), del(17p), and trisomy 12 assessment, *TP53* mutational analysis, immunoglobulin gene heavy chain variable (*IGHV)* region somatic hypermutation (SHM) analysis and B-cell receptor stereotype subset analysis (subset #2 configuration) are all essential for full prognostic evaluation of CLL/SLL [39–41]. Detection of karyotypic complexity and *BTK, PLCG2*, and *BCL2* mutation status all remain desirable additional investigations in the context of targeted therapy. *IGHV* mutation and *TP53* aberration status are both included in the CLL-international prognostic index (CLL-IPI) [42], along with age, clinical stage and beta 2-microglobulin level. The International Prognostic Score for early-stage CLL/SLL (IPS-E) includes *IGHV* mutation status, absolute lymphocyte count >15 × 10^9^/L, and presence of palpable lymph nodes [43]. In the setting of transformation, use of the term “Richter transformation” is recommended over “Richter Syndrome”.

B-prolymphocytic leukaemia (B-PLL) of WHO-HAEM4R is *no longer recognized* in WHO-HAEM5 in view of its heterogeneous nature. Cases that have been labeled as B-PLL include: (1) a variant of mantle cell lymphoma, characterized by presence of *IGH*::*CCND1;* (2) prolymphocytic progression of CLL/SLL, defined by CD5-positive non-mantle B-cell neoplasm with >15% prolymphocytes in the peripheral blood and/or bone marrow [44–47], and (3) other cases, now classified as “splenic B-cell lymphoma/leukaemia with prominent nucleoli”.

### Splenic B-cell lymphomas and leukaemias: The term “splenic B-cell lymphoma/ leukaemia with prominent nucleoli” replaces “hairy cell leukaemia variant” and “CD5-negative B-cell prolymphocytic leukaemia”

**Fig. 1:**
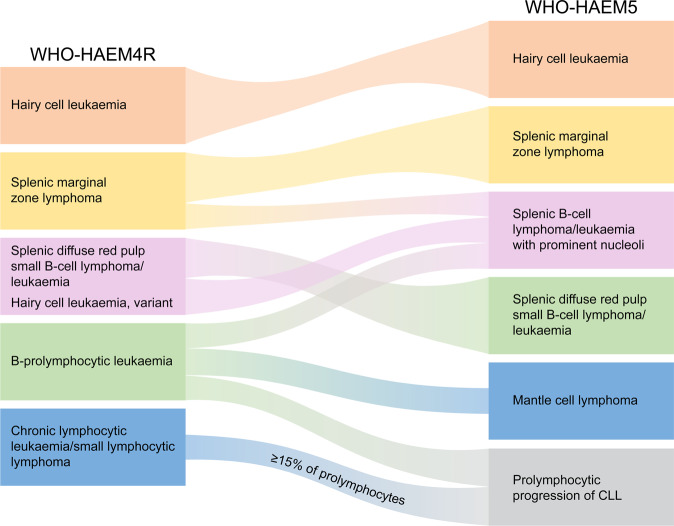
Summary of the relationship between splenic B-cell lymphoma entities as named and defined in the revised 4^th^ edition of the WHO classification (WHO-HAEM4R) and in the present 5^th^ edition (WHO-HAEM5). Some cases previously classified as B-prolymphocytic leukaemia do represent (blastoid) mantle cell lymphoma (as was already indicated in WHO-HAEM4R) or prolymphocytic progression of CLL. Cases classified in WHO-HAEM4R as CLL/SLL with ≥ 15% of prolymphocytes are now classified as prolymphocytic progression of CLL, cases with <15% of prolymphocytes remain CLL/SLL in WHO-HAEM5. Remaining cases are now renamed as “splenic B-cell lymphoma/leukaemia with prominent nucleoli” (SBLPN). This latter entity has absorbed cases formerly classified as hairy cell leukaemia variant (HCLv) and very rare cases of splenic marginal zone lymphoma with similar morphological features. It should be noted that the distinction between the various entities cannot always be made in the absence of a splenectomy specimen.

The splenic B-cell lymphoma and leukaemia family in WHO-HAEM5 includes hairy cell leukaemia (HCL), splenic B-cell lymphoma/leukaemia with prominent nucleoli (SBLPN), splenic diffuse red pulp small B-cell lymphoma (SDRPL) and splenic marginal zone lymphoma (SMZL) (Fig. 1). In contrast to WHO-HAEM4R, SBLPN and SDRPL are now separately classified, with a nomenclature change in the former. **Hairy cell leukaemia** is a mature B-cell neoplasm with distinctive clinicopathologic features and BRAF p.V600E (NP_004324.2) somatic mutation in ≥95% of cases [48]. Other splenic small B-cell lymphomas usually lack *BRAF* mutations.

The new entity **splenic B-cell lymphoma/leukaemia with prominent nucleoli (SBLPN)** replaces the previous term “hairy-cell leukaemia variant“, in recognition that this proliferation is biologically distinct from HCL, although the leukaemic cells may partly resemble the “hairy cells” of HCL. Moreover, this entity also absorbs all cases previously termed CD5-negative B-prolymphocytic leukaemia (B-PLL) per WHO-HAEM4R. Although data from the literature cannot be directly extrapolated to the new class, it can be stated that SBLPN is rare, comprising approximately 0.4% of chronic lymphoid malignancies [49–53], and affects mainly elderly patients. The neoplastic cells have prominent nucleoli and are negative for HCL markers CD25, annexin A1, TRAP, and CD123. SBLPN is clinically more aggressive than HCL and resistant to cladribine as single-agent treatment. More recently improved sensitivity to cladribine in combinations with rituximab or bendamustine has been shown [49, 54–56].

**Splenic diffuse red pulp small B-cell lymphoma (SDRPL)** has some features overlapping with HCL and SBLPN but can be distinguished on careful evaluation of morphologic and immunophenotypic characteristics. A CD200 mean fuorescence intensity (MFI)/CD180 MFI ratio <0.5 on flow cytometry favours a diagnosis of SDRPL over HCL, SMZL, and SBLPN [57]. These entities can be best discriminated by pathologic examination of the spleen; in the absence of a splenectomy specimen, bone marrow examination shows characteristic features in SDRPL with a predominant intrasinusoidal pattern, while SMZL and SBLPN have a more diverse growth pattern in the bone marrow and HCL shows a typical diffuse pattern with reticulin fibrosis [58, 59]. In absence of a splenectomy specimen, however, the distinction is often not possible.

### Lymphoplasmacytic lymphoma: IgM matters

WHO-HAEM5 recognizes two subtypes of **lymphoplasmacytic lymphoma (LPL)**, the most common being the IgM-LPL/Waldenström Macroglobulinaemia (WM) type. Non-WM type LPL represents around 5% of LPL and includes: (1) cases with IgG or IgA monoclonal proteins, (2) non-secretory LPL, and (3) IgM LPL without bone marrow involvement [60–65].

There are two molecular subsets of IgM-LPL/WM type based on the presence or absence of the MYD88 p.L265P (NP_002459.2) mutation, which is regarded as the hallmark driver mutation in the vast majority of LPL (>90%) [66–69]. Demonstration of the MYD88 p.L265P mutation may aid in the difficult differential diagnosis with nodal and extranodal marginal zone lymphomas (MZL) with plasmacytoid differentiation and plasma cell (multiple) myeloma. The two latter entities generally lack the MYD88 p.L265P mutation with the exception of rare cases of MZL. *CXCR4* mutations occur in up to 40% of all LPLs, usually concurrent with *MYD88* mutations. It is desirable to perform *CXCR4* mutational analysis for patients considered for treatment with a BTK inhibitor, since this genetic context is not only associated with shorter time to treatment, but especially with resistance to ibrutinib therapy [70].

### Marginal zone lymphomas: cytogenetic and mutational profiles differ by anatomic site, and cutaneous MZL achieves independence

**Extranodal marginal zone lymphoma of mucosa-associated lymphoid tissue (EMZL)** and **nodal marginal zone lymphoma (NMZL)**, featured as distinct entities in WHO-HAEM4R, are retained in WHO-HAEM5. **Paediatric nodal marginal zone lymphoma** (pNMZL) is upgraded from a subtype under nodal marginal zone lymphoma to a separate entity. Although it shows overlapping features with paediatric-type follicular lymphoma, current published evidence is considered insufficient to group these two indolent paediatric diseases into one family at this time. **Primary cutaneous marginal zone lymphoma (PCMZL)** has also been designated as a separate entity in WHO-HAEM5, owing to its distinctive clinicopathologic features.

EMZL, NMZL, and PCMZL have overlapping histologic and immunophenotypic features: the neoplastic cells are mature small B cells typically negative for CD5 and CD10. Plasmacytic differentiation is common, and associated reactive lymphoid follicles are often present. However, despite some shared features, they have different etiologies and pathogenesis, with further differences among EMZLs arising in different anatomic sites. Trisomy of chromosomes 3 and 18 are common in all. Gains of chromosomes 2p and 6p, and loss of 1p and 6q are frequent in NMZL [71–77]; however, gain of 6p and loss of 6q are recurrently seen only in EMZL of the ocular adnexa [78]. Translocations involving *MALT1* such as t(11;18)(q21;q21), resulting in *BIRC3*::*MALT1* fusion, are recurrent in gastric and pulmonary EMZL but rare at other sites [79–83]. In contrast, no recurrent gene fusions or rearrangements are described in PCMZL or NMZL.

**Fig. 2:**
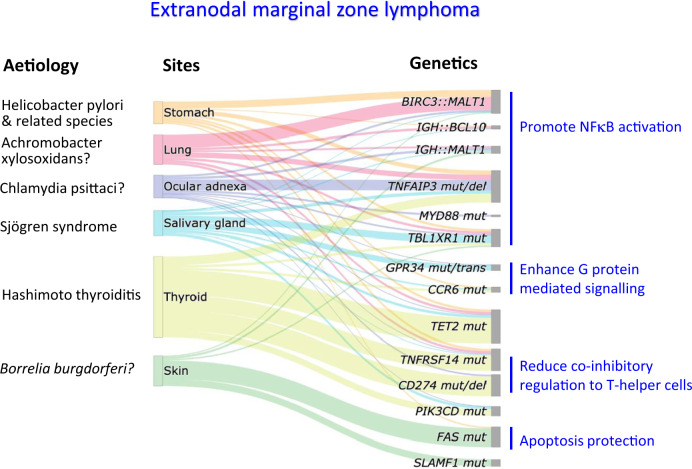
Aetiology and recurrent genetic abnormalities in extranodal marginal zone lymphoma (EMZL) of various sites. An important clinical application is that *BIRC3*::*MALT1* identifies those cases of the gastric EMZL not responding to *H. pylori* eradication. As many of the genes involved in EMZL have not been uniformly investigated across different sites, only the recurrent genetic changes fundamental to the understanding of EMZL pathogenesis are presented. The height of the boxes under sites does not reflect the frequencies of these lymphomas. trans translocation, mut mutation, del: deletion.

The mutational profiles of EMZL and NMZL differ [76, 84–86]. In addition, there are significant genetic differences among EMZLs arising in different anatomic sites (Fig. 2): e.g., ocular adnexal EMZL commonly shows *TNFAIP3* mutation/deletion [87, 88]; salivary gland EMZL shows recurrently mutated *GPR34* [89, 90]; most thyroid EMZL carry deleterious mutations of *CD274*, *TNFRSF14* and/or *TET2* [91]; and PCMZL often shows *FAS* mutations [92]. Somatic variants of *KMT2D*, *PTPRD*, *NOTCH2, KLF2*, and others are frequent in NMZL [76, 84, 85, 93] but not in EMZL. Better definition of the underlying molecular genetic changes of these lymphomas may potentially open the door to improved treatment options.

### Follicular lymphoma (FL): from classic grading to biological grouping

The family of follicular lymphoma encompasses follicular lymphoma, in situ follicular B-cell neoplasm (ISFN), paediatric-type FL and duodenal-type FL. There are no significant updates on the latter three entities in WHO-HAEM5. In contrast, the entity of **follicular lymphoma** has undergone significant revision. The vast majority of FL (85%) have at least in part a follicular growth pattern, are composed of centrocytes and centroblasts and harbour the t(14;18)(q32;q21) translocation associated with *IGH*::*BCL2* fusion; these are now termed **classic FL (cFL)** and set apart from two related subtypes/groups, **follicular large B-cell lymphoma (FLBL**) and **FL with uncommon features (uFL)**.

In WHO-HAEM5, grading of FL, which is only pertinent to cFL, is no longer mandatory. This decision was made after extensive discussions and evaluation of the literature centering on the reproducibility of grading and on its questionable clinical significance for individual patients in the era of modern therapy. Poor reproducibility may result from various causes, including sampling (complete lymph node excision versus core needle biopsy), definition and recognition of centroblasts, and methods of enumeration. Since grading of FL is based on the enumeration of centroblasts per high-power field (HPF), one of the challenges is the lack of a consistent definition of a HPF using a 40x microscope objective (400x magnification), where the size of the microscopic field has changed over the years even at the same magnification [94]. Lack of consensus regarding the morphological spectrum of centroblasts and using conventional methods of counting further negatively impacts reproducibility [95]. Clinical outcomes among patients with FL of grades 1, 2, and 3A seem not to be significantly different. Currently, patients are treated with similar protocols both in and outside clinical trials in many parts of the world [96–99]. While attempts have been made to improve reproducibility through digital applications or by using immunohistochemical supportive data, such methods have not been compared to patient outcome. Hence, it was deemed premature to include them in WHO-HAEM5 [100–102]. Taken together, for histopathologic as well as clinical reasons, it was felt timely to make grading of FL to be optional in the subtyping of cFL.

Rare cases of cFL grade 3A may show a focal or extensive diffuse growth pattern. In WHO-HAEM4R, the recommended diagnosis in such cases was “DLBCL with follicular lymphoma”, even though sheets of large cells are not often present. Currently, it is uncertain whether such cases should better be classified as cFL or DLBCL [103] and therefore, treatment decisions in individual patients should not be based on pathology information alone but rather be made in multidisciplinary conference settings and await research to define more objective criteria to predict clinical course. The subtype of FLBL largely equals WHO-HAEM4R FL grade 3B, and renaming was done for reasons of consistency throughout the classification.

The newly introduced subtype of uFL includes two subsets that significantly diverge from cFL: one with “blastoid” or “large centrocyte” variant cytological features, and the other with a predominantly diffuse growth pattern [104, 105]. FL with “blastoid” or “large centrocyte” cytological features more frequently display variant immunophenotypic and genotypic characteristics and may show inferior survival [106]. They need to be distinguished from large B-cell lymphoma with *IRF4* rearrangement [107]. FL with a predominantly diffuse growth pattern frequently occurs as a large tumour in the inguinal region and is associated with CD23 expression, an absence of *IGH*::*BCL2* fusion [108], and frequent *STAT6* mutations along with 1p36 deletion or *TNFRSF14* mutation [104, 109]. Separating such cases from cFL will support research to clarify disease biology, allowing a better definition in future classifications.

### Mantle cell lymphoma: Improved risk stratification

WHO-HAEM5 groups mantle cell neoplasia into three individual chapters. ***In situ*** **mantle cell neoplasm (ISMCN)** is rare and typically an incidental finding. It represents colonization of mantle zones of lymphoid follicles by B cells carrying an *IG*::*CCND1* fusion leading to cyclin D1 overexpression [110].

The *IGH*::*CCND1* fusion associated with t(11;14)(q13;q32) is the genetic hallmark of **mantle cell lymphoma (MCL)**, present in ≥95% of cases (i.e., cyclin D1-positive MCL subtype) [111, 112]. Occasionally, *IGK* or *IGL* serve as the *CCND1* translocation partner [113]. In the occasional cases of MCL that strongly express cyclin D1 protein but show no *CCND1* rearrangement by FISH, genomic studies have revealed cryptic rearrangements of *IGK* or *IGL* enhancers with *CCND1* [114–116]. In the small subset of MCL negative for cyclin D1 expression and *CCND1* rearrangement (i.e., cyclin D1-negative MCL subtype), *CCND2, CCND3*, or *CCNE* rearrangements have been identified as alternative mechanisms of cell cycle dysregulation [117]. In recent years, the median overall survival of patients with MCL has dramatically increased due to improved therapies. Hence, the identification of prognostic subgroups has become highly relevant. Widely available and best-established biomarkers of high-risk MCL include cytomorphology (pleomorphic or blastoid appearance), high Ki67 proliferative index, p53 expression and *TP53* mutation [118, 119].

**Non-nodal MCL (nnMCL)** is characterized by involvement of blood, bone marrow and spleen, little or no lymphadenopathy, a mostly asymptomatic presentation, and a better clinical outcome compared to MCL. Biologically, nnMCL differs from MCL by: (i) lack of SOX11 expression [120, 121], low Ki67 index and frequent lack of CD5 expression [122]; (ii) differences in the usage of *IGHV* gene segments with biased usage of the *IGHV1-8* gene [122] together with a higher somatic hypermutation load [121, 123, 124]; and (iii) fewer genetic alterations and infrequent genomic complexity [120, 125].

### High-grade transformation steps forth

For the first time, WHO-HAEM5 now includes a section with the description of **High-grade transformation of indolent B- cell lymphomas** including a summary of the incidence of known and driver genes.

### Large B-cell lymphomas: new names and new umbrellas

The family of **large B-cell lymphomas** comprises a wide spectrum of tumours. Although these are generally composed of medium-sized to large cells with round to ovoid nuclei and vesicular chromatin, cases with intermediate-sized and blastoid cells may also meet criteria for this family. These require delineation from morphologically similar entities, such as the blastoid variant of mantle cell lymphoma and lymphoblastic leukaemia/lymphoma.

**Diffuse large B-cell lymphoma, not otherwise specified (DLBCL, NOS)** represents the most common entity, and is defined by large-cell morphology as above, mature B-cell phenotype, and lack of criteria defining specific large B-cell lymphoma entities. The lymphomas encompassed within DLBCL, NOS are morphologically and molecularly heterogeneous. Since most DLBCL, NOS broadly recapitulate the differentiation and maturation mechanisms active in germinal centers (GC), two main subtypes previously defined in WHO-HAEM4R continue to be recognized. The germinal centre B-cell-like (GCB) subtype has a gene expression profile (GEP) related to a GC cell of origin (COO), and is enriched for *IGH*::*BCL2* fusion due to t(14;18)(q32;q21) and mutations of genes instrumental for GC development, GC dark zone and light zone transitions and microenvironmental interactions, such as *EZH2, GNA13, MEF2B, KMT2D, TNFRSF14, B2M* and *CREBBP* [126]. The activated B-cell-like (ABC) subtype derives from cells of GC exit or post GC origin, with either germinal center-exit or early plasmablastic phenotype. It is characterized by dependence on BCR signaling and NFκB activities, is negative for most GC markers, and expresses IRF4/MUM1 [127]. It is enriched for BCR pathway mutations such as in *MYD88* (mostly p.L265P), *CD79B* and *PIM1*, as well as genetic changes that block the B-cell differentiation program such as *BCL6* rearrangements and *PRDM1/BLIMP1* mutation/deletion [126]. It is recommended to continue rendering the GCB/ABC (GCB/nonGCB) distinction although it has become apparent that the clinical impact of COO stratification is relatively limited outside clinical trials. Although IHC algorithms obviously do not recognize the “unclassified” GEP category and have concordance issues with GEP, they are widely used in routine practice. Recent data from next generation sequencing studies have illustrated a heterogeneous molecular landscape of DLBCL, NOS with around 150 genetic drivers that are recurrently mutated in DLBCL, with a mean of approximately 8% of these genes mutated per patient [128]. Interestingly, in spite of the use of various sequencing approaches and clustering algorithms, the genetic landscape of DLBCL, NOS can be used for sub-classification with broad concordance suggesting that the underlying disease biology can be captured by mutational analysis. Some of the genetic groups harbour a mutational profile that in part overlaps with those of FL or MZL, suggesting either transformation from these low-grade lymphomas or a common path in their early pathogenesis. However, no unifying concept for proposed clusters and the significance of their genetic drivers has been established so far, precluding the definition of a unified genetic framework of DLBCL, NOS at the present time. Moreover, the impact of these genetic clusters on outcome and as a basis for targeted treatment approaches is currently unclear and awaits evidence from clinical trials. Therefore, it was considered premature to introduce such molecular classifications in WHO-HAEM5.

**Fig. 3:**
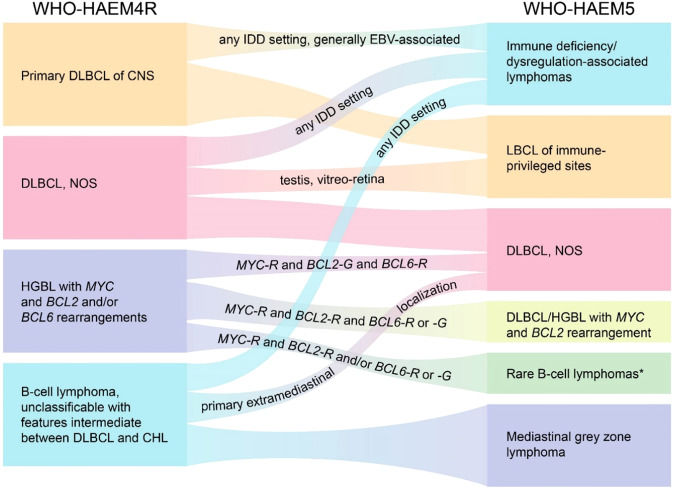
Summary of the relationship between large B-cell lymphoma (LBCL) entities as named and defined in the revised 4^th^ edition of the WHO classification (WHO-HAEM4R) and in the present 5^th^ edition (WHO-HAEM5). * “Rare B-cell lymphomas” refer to those fulfilling definitions of specific clinico-pathological entities while incidentally bearing concomitant *MYC* and *BCL2* rearrangements. Examples are fluid-overload-associated large B-cell lymphomas and rare follicular lymphomas. R rearrangement, G germline configuration.

WHO-HAEM5 recognizes 17 specific entities as “large B-cell lymphomas” other than DLBCL, NOS (Table 1 and Fig. 3**)**. For most of these entities, biological concepts and diagnostic strategies have remained largely unchanged compared with WHO-HAEM4R. However, the names of some entities have been modified for reasons of consistency, from “diffuse large B-cell lymphoma“ to “large B-cell lymphoma“, acknowledging the fact that a diffuse growth pattern is either not apparent/present or cannot be assessed in some entities (e.g., fibrin-associated large B-cell lymphoma or fluid-overload associated large B-cell lymphoma).

**Fig. 4:**
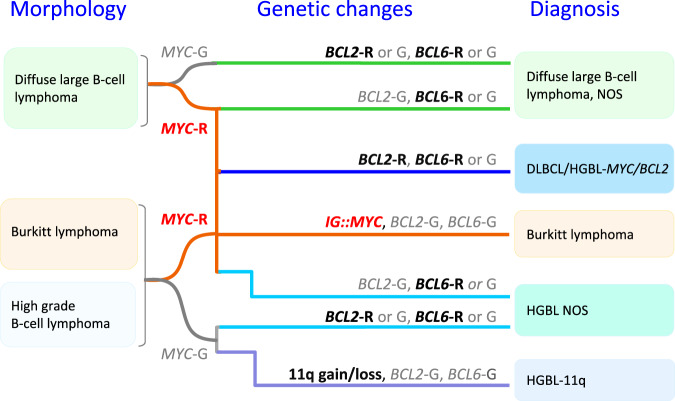
Algorithm for classification of aggressive B-cell lymphomas in WHO-HAEM5 in the light of *MYC*, *BCL2* and *BCL6* rearrangement and complex 11q gain/loss patterns. HGBL high grade B-cell lymphoma, R rearrangement, G germline configuration.

The WHO-HAEM4R entity of high-grade B-cell lymphoma with dual rearrangements of *MYC* and *BCL2* and/or *BCL6* has been conceptually reframed and reassigned. In recognition of their variable morphologies but homogeneous dark zone biologic features and gene expression characteristics, the WHO-HAEM5 renames the entity **diffuse large B-cell lymphoma/high-grade B-cell lymphoma with** ***MYC*** **and** ***BCL2*** **rearrangements** (DLBCL/HGBL-*MYC/BCL2*) to encompass tumours defined by the presence of dual *MYC* and *BCL2* rearrangements that may be composed of large or intermediate or blastoid cells (Fig. 4). Hence, the primary morphological categorization of the neoplasm can be maintained after determining the genetic constitution. This group of cases forms a homogeneous entity with an exclusive GC gene expression profile, a close pathogenetic relationship to FL and molecular GC-like DLBCL subsets [129–132]. In addition, gene expression signatures associated with DLBCL/HGBL-*MYC/BCL2 (MHG, DHITsig)* [130, 133] significantly overlap with those of Burkitt lymphoma (BL). In contrast, lymphoid neoplasms with dual *MYC* and *BCL6* rearrangements represent a more diverse spectrum [129] with variable gene expression profiles and mutational spectra, markedly differing from DLBCL/HGBL-*MYC/BCL2*. Hence, these cases have been excluded from the DLBCL/HGBL-*MYC/BCL2* entity and are now classified either as a subtype of DLBCL, NOS or HGBL, NOS according to their cytomorphological features (Fig. 4).

**High-grade B-cell lymphoma with 11q aberration (HGBL-11q)**, formerly known as Burkitt-like lymphoma with 11q aberration in WHO-HAEM4R, is an aggressive *MYC* rearrangement-negative mature B-cell lymphoma with a morphology similar to Burkitt lymphoma (BL) or with an intermediate or blastoid appearance, an immunophenotype (CD10+, BCL6+, BCL2-), and/or gene expression profile (GEP) similar to BL, and a characteristic chromosome 11q-gain/loss pattern. The losses in 11q24qter are more specific to this entity than the centromeric gains but rarely might be substituted by copy-number neutral losses of heterozygosity. More recent studies have also confirmed that the mutational spectrum, besides the pattern of genomic imbalances, is different from that of BL and is more similar to that of DLBCL of GCB type. Of note, genomic alterations affecting the ID3-TCF3 complex, one of the molecular hallmarks of BL, are only rarely, if at all, seen in HGBL-11q [134, 135]. Cases of B-cell lymphoma with a Burkitt-like appearance that lack *MYC* rearrangement, therefore, should be tested for the 11q gain/loss pattern [136] (Fig. 4). It should be noted that the morphological spectrum of HGBL-11q as defined by the specific 11q-gain/loss pattern is more restricted than that of DLBCL/HGBL-*MYC/BCL2*.

**Large B-cell lymphomas (LBCL) of immune-privileged sites** is a new umbrella term introduced in WHO-HAEM5 to acknowledge common biological features of a group of aggressive B-cell lymphomas that arise as primary tumours in the central nervous system (CNS), the vitreoretinal compartment, and the testes of immunocompetent patients. This new entity now combines the previous entity of primary DLBCL of CNS with DLBCL of the vitreoretina and testis that were previously included among DLBCL, NOS. They arise in immune sanctuaries created by their respective anatomical structures (e.g., the blood-brain, blood-retinal, and blood-testicular barriers), and immune regulation systems within their respective primary sites, and share immunophenotypic and molecular features [137–139] (Table 4). Information on this group of tumours is rapidly accruing: it appears that some lymphomas arising at other distinct sites such as the breast and skin share some of these features, and thus, this group of ‘immune-privileged lymphomas’ might expand in future classifications.

**Table 4:** Distinctive features of primary large B-cell lymphomas of immune privileged sites.

Subtypes	Primary large B-cell lymphoma of the CNS
	Primary large B-cell lymphoma of the vitreoretina
	Primary large B-cell lymphoma of the testis
Clinical	Usually in adults over age of 60 years
	Lymphoma tends to “home” to other immune privileged sites: vitreoretina tumour may occur concurrently with or follow CNS tumour; testicular tumour tends to relapse in CNS or contralateral testis
	Aggressive tumours with generally poor prognosis
Morphology	Large B-cell lymphoma
Immunophenotype	Activated B-cell immunophenotype: Usually CD10-, MUM1+, BCL6+
	EBV negative
Mutational profile	Concomitant *MYD88* and *CD79B* mutations
	Immune evasion: genetic inactivation of MHC class I and II and *B2M* (β _2_-microglobulin) with subsequent loss of protein expression
	Showing DLBCL genomic signature C5/MCD/MYD88

**Fluid overload-associated large B-cell lymphoma** is a new addition to the list of large B-cell lymphomas in WHO-HAEM5, being distinct from primary effusion lymphoma (PEL). This entity has been briefly alluded to in the 5^th^ Edition of the WHO Classification of Thoracic Tumours, under the names “PEL-like lymphoma” or “HHV8-unrelated PEL-like lymphoma” [140]. Patients usually are adults, predominantly elderly, without underlying immunodeficiency, who present with exclusive involvement of body cavities, most commonly the pleural cavity [141–143]. They frequently have an underlying condition causing fluid overload, such as chronic heart failure, renal failure, protein-losing enteropathy or liver failure/cirrhosis. The neoplastic large cells exhibit a mature B-cell rather than plasmablastic immunophenotype. KSHV/HHV8 is negative, while EBV is positive in 13–30% of cases and the genomic landscape differs essentially from primary effusion lymphoma (PEL) [141, 142]. The prognosis appears to be fairly favorable, yet another reason for distinction from PEL.

**Mediastinal gray zone lymphoma (MGZL)** is a B-cell lymphoma with overlapping features between primary mediastinal B-cell lymphoma (PMBL) and classic Hodgkin lymphoma (CHL), especially nodular sclerosis CHL (NSCHL). This entity replaces the term “B-cell-lymphoma, unclassifiable with features intermediate between DLBCL and classic Hodgkin lymphoma” of the WHO-HAEM4R, taking into account that lymphomas with these features are specific to the mediastinum and are part of a single biologic group with a morphologic and immunophenotypic spectrum from CHL to PMBL, with MGZL straddling the two. Current evidence indicates that cases with morphologic and immunophenotypic features similar to MGZL, but occurring outside and without involvement of the mediastinum, harbour different gene expression profiles and DNA alterations [143]. Hence, these cases are better classified as DLBCL, NOS.

**High grade B-cell lymphoma, NOS** (HGBL, NOS) represents aggressive mature B-cell lymphomas composed of medium-sized or blastoid cells that do not fit into other well-defined entities. NGS-based analyses of the mutational spectrum and gene expression signatures suggest that HGBL, NOS is a heterogeneous category, also including activated B-cell lymphomas with mutations of *MYD88, CD79B*, or *TBL1XR1*. Most frequent mutations are found in *KMT2D* (43%) and *TP53* (30%). By GEP, most cases of HGBL, NOS have been reported to group into the “unclassified” cluster, and the remainder are variably classified in the other clusters [144]. Interestingly, gene expression profiling showed that 54% of HGBL, NOS harbour the “double hit” signature (DHITsig) characteristic of LBCL/HGBL with *MYC/BCL2* despite lacking rearrangements of these genes [144].

### Burkitt lymphoma: EBV matters

The definition of **Burkitt lymphoma (BL)** in WHO-HAEM5 remains largely unchanged, describing BL as an aggressive mature B-cell neoplasm composed of medium-sized cells with a germinal center B-cell phenotype CD10+, BCL6+, BCL2-/weak, high Ki67 index (>95%) and an *IG*::*MYC* juxtaposition (Fig. 4). Whereas three subtypes of BL have been historically recognized (“endemic”, “non-endemic or sporadic”, and “immunodeficiency-associated”) [145], more recent data suggest that EBV-positive BL and EBV-negative BL form discrete biologic groups based on their molecular features regardless of epidemiologic context and geographic location and therefore supersede the epidemiological subtyping [146–151]. EBV infection plays an essential role early in pathogenesis causing B cells to evade apoptosis [152, 153]. Emerging evidence suggests a dual mechanism of BL pathogenesis: virus-driven versus mutational, depending on EBV status [147]. EBV-positive and EBV-negative BL share evidence of coding mutations affecting pathways such as BCR and PI3K signaling, apoptosis, SWI/SNF complex and GPCR signaling [149, 154, 155]. In comparison with EBV-negative BL, EBV-positive BL shows significantly higher levels of somatic hypermutation particularly in noncoding sequences close to the transcription start site [149], harbours fewer driver mutations, particularly in the apoptosis pathway [149], and shows a lower frequency of mutations in the genes encoding the transcription factor TCF3 or its repressor ID3 [149]. To acknowledge these recent insights into BL biology, the distinction of the two subtypes, EBV-positive BL vs. EBV-negative BL, is recommended by WHO-HAEM5.

### KSHV/HHV8-associated B-cell lymphoid proliferations and lymphomas

WHO-HAEM5 recognizes the full spectrum of lymphoid proliferations related to Kaposi sarcoma herpesvirus/human herpesvirus 8 (KSHV/HHV8) infection, which in parallel with the terminology for other herpesviruses is now indicated as KSHV/HHV8 to accommodate both the common practices of haematopathologists and of virologists. These lymphoid proliferations include **KSHV/HHV8-associated multicentric Castleman disease (KSHV/HHV8-MCD)** [covered under the category “Tumour-like lesions with B-cell predominance”], **germinotropic lymphoproliferative disorder (KSHV/HHV8-GLPD)**, **primary effusion lymphoma (PEL)**, **extracavitary PEL (EC-PEL)** and **KSHV/HHV8-positive diffuse large B-cell lymphoma (KSHV/HHV8-DLBCL)** [156, 157]. PEL/EC-PEL and KSHV/HHV8-DLBCL are characteristically seen in HIV patients, but can be seen in other immunodeficiency settings. KSHV/HHV8-GLPD, in contrast, is more prevalent in elderly patients without overt immunodeficiency, although it has also been reported in HIV-positive individuals. In addition, KSHV/HHV8-MCD is seen in both HIV-positive and HIV-negative patients, but the latter are overall older [158–160]. The diagnosis of prototypical examples of the various KSHV/HHV8-associated entities is often straightforward based on the definitions as formulated in WHO-HAEM5. It has become clear, however, that the morphological and clinical spectrum of KSHV/HHV8-associated entities is broader than previously appreciated [161]. Moreover, there are individual patients in whom clinical, histologic and viral features (KSHV/HHV8 with or without EBV) overlap among entities. Observations of synchronous and metachronous presentation of different KSHV/HHV8-associated lesions, and cases that possess morphological and/or clinical features of more than one entity support the notion that these equivocal cases may result from the special biology of KSHV/HHV8, which is not adequately captured by current disease-defining criteria [158, 161, 162]. For example, distinction between lymph node-based extracavitary PEL and KSHV/HHV8-positive DLBCL is difficult and may be arbitrary. WHO-HAEM5 acknowledges the limitations of its definitions. While more data to support biology-defined boundaries among the entities are awaited, it is recommended that decisions on classification and optimal therapy should be resolved in a multidisciplinary setting in challenging cases.

### Lymphoid proliferations and lymphomas associated with immune deficiency and dysregulation: a new approach to order patterns

**Fig. 5:**
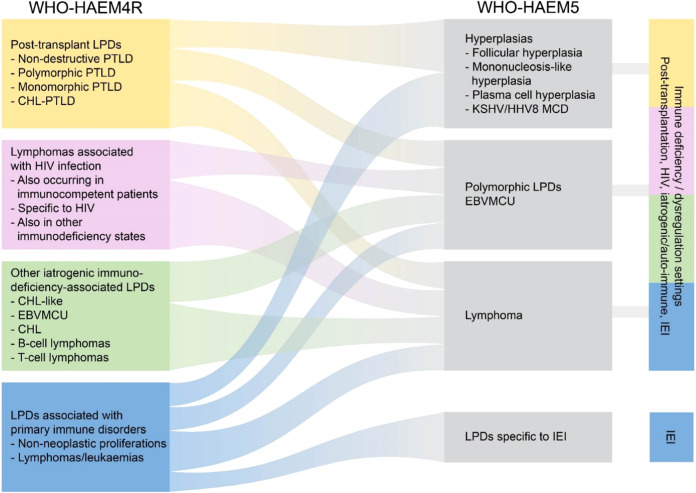
Summary of the relationship between immunodeficiency-associated lymphoid proliferations and lymphomas as named and defined in the revised 4^th^ edition of the WHO Classification (WHO-HAEM4R) and in the present 5^th^ edition (WHO-HAEM5). The overarching concept applied in WHO-HAEM5 recognizes the pathological and biological similarities between proliferations presenting in various immune deficiency settings, while acknowledging their specific features. Outside the shared entities, unique proliferations are especially typical for various inborn errors of immunity (IEI). EBVMCU: EBV-positive mucocutaneous ulcer.

WHO-HAEM5 has introduced major changes to the classification of immunodeficiency-associated lymphoproliferative disorders (Fig. 5). In prior classifications, these disorders were grouped according to the disease background in which they arose and were discussed in separate chapters: primary immunodeficiencies, HIV infection, post-transplantation and other iatrogenic immunodeficiencies. This approach has been valuable for many years in supporting clinical decision-making and as a basis for translational and basic research. Knowledge that has resulted from this approach supports the notion that morphological features and, to a certain extent, the biology of many of these entities overlap and that the spectrum of immunodeficiency settings is broader than previously recognized. Therefore, it was considered timely to introduce an overarching framework and a standardized nomenclature to cover the different settings of immune dysfunction, according to the unifying nomenclature proposed at the Workshop on Immunodeficiency and Dysregulation organized by the Society of Hematopathology and European Association for Haematopathology in 2015 [163]. This framework aims to focus attention on shared histologic and pathogenetic features as well as to accommodate distinct causal associations of specific lesions and specific clinical and/or therapeutic consequences [163, 164]

The new standardized nomenclature builds on an integrated approach to diagnosis that combines all relevant data into a reporting system as follows (Table 5):

**Table 5:** Three-part nomenclature for lymphoid proliferations and lymphomas arising in the setting of immune deficiency/dysregulation.

Histological diagnosis	Viral association	Immune deficiency/dysregulation setting
◦ Hyperplasia (specify type) ◦ Polymorphic lymphoproliferative disorder ◦ Mucocutaneous ulcer ◦ Lymphoma (classify as for immunocompetent patients)	◦ EBV +/− ◦ KSHV/HHV8 +/−	◦ Inborn error of immunity (specify type) ◦ HIV infection ◦ Posttransplant (specify: solid organ/bone marrow) ◦ Autoimmune disease ◦ Iatrogenic/therapy-related (specify) ◦ Immune senescence

1) Histological diagnosis according to accepted criteria and terminology;

2) Presence or absence of one or more oncogenic virus(es); and

3) The clinical setting/immunodeficiency background.

This nomenclature addresses existing inconsistencies in terminology and diagnostic criteria for similar lesions in different immunodeficiency settings, may improve communication among multidisciplinary teams in guiding appropriate clinical management as well as research studies, and facilitate incorporation of emerging knowledge in the field. As the same pathological entity, e.g., polymorphic lymphoproliferative disorder, does not necessarily have the same pathogenesis or clinical behaviour in different immune deficiency/dysregulation settings, this underscores the need to include the immune deficiency/dysregulation setting as a required part of the three-part nomenclature.

New types of immunodeficiency settings continue to be recognized [165–167]. Poly-chemotherapy for treatment of solid tumours and haematologic neoplasms has been largely accepted as an underlying cause for immunodeficiency. However, it is as yet unclear which polychemotherapy regimens confer this risk, and how long the risk persists [168]. In addition, with increasing use of novel immune modulatory agents, unanticipated types of immune dysfunction are emerging, e.g., in the aftermath of CAR-T cell and/or checkpoint inhibition therapies. Immune senescence is another setting that is poorly understood and as yet not possible to define or exclude; thereby use of an arbitrary age as cut-off does not have a scientific basis [169]. All these emerging concepts call into question the adequacy of the term “immunodeficiency”, which does not capture the extent, depth or phenotypic variation of immune suppression and the milieu of deregulated immune cell subsets. Therefore, WHO-HAEM5 has adopted “immune deficiency/dysregulation” (IDD) as the preferred term to encompass this expanding disease spectrum.

Primary immunodeficiencies, associated with germline mutations, have been renamed “inborn errors of immunity” (IEI) by the International Union of Immunological Societies, a terminology adopted by WHO-HAEM5 [170]. Patients with IEI may develop distinctive types of lymphoid proliferations unique to the particular IEI, as well as those described in the acquired IDD settings. The types and frequency of these proliferations are largely dependent on the immune dysregulation conferred by the germline aberration underyling a respective IEI. Given the overlap with other IDD settings, IEI-associated lymphoid proliferations and lymphomas have been incorporated into the overarching framework and nomenclature of “lymphoid proliferations and lymphomas associated with immune deficiency and dysregulation.”

The new approach to the classification of IDD-associated lymphoid proliferations and lymphomas impacts other lymphoid entities that are described in separate WHO chapters. This especially holds true for diagnoses in which EBV plays a defining or important role, including EBV-positive DLBCL, lymphomatoid granulomatosis, and CHL. In WHO-HAEM5, harmonization of diagnostic criteria among these categories has been undertaken as much as currently feasible, while at the same time acknowledging that some terminologies are arbitrary. For example, should an elderly patient with a DLBCL harbouring EBV be diagnosed as having EBV+ DLBCL or DLBCL, EBV+, in an IDD setting based upon presumed immune senescence? Clarification of these disease boundaries awaits further clinico-pathological data and further insights into disease pathogenesis, which will allow evidence-based refinements to the classification.

### Hodgkin lymphoma: CHL clearly defined from its mimickers, NLPHL on the way to, but not yet NLPBCL

**Classic Hodgkin lymphoma (CHL)** comprises a group of B-cell neoplasms derived from germinal center B-cells, characterized by a low number of tumour cells embedded in a reactive microenvironment rich in immune cells. The large diagnostic Hodgkin and Reed-Sternberg (HRS) cells characteristically show a defective B-cell program. The defining immunophenotype of HRS cells remains unchanged from WHO-HAEM4R, as are criteria for nodular sclerosis (NSCHL), mixed cellularity (MCCHL), lymphocyte rich (LRCHL), and lymphocyte depleted (LDCHL) subtypes. With modern treatment protocols, these subtypes have lost most of their prognostic relevance. However, there is still merit in describing these subtypes to support epidemiological and translational studies, since specific subtypes are associated with different clinical features and underlying biologies [171]. While the basic description has not changed substantially since the last century, WHO-HAEM5 includes a comprehensive section on the etiology and pathogenesis of CHL, in particular incorporating new data on the crucial role of the microenvironment in modulating the disease [172, 173]. Recent biological insights have led to the recognition of an expanding spectrum of pitfalls, grey zones and mimickers, among them nodal T follicular helper cell lymphomas and lymphoproliferative disorders arising in immune deficiency/dysregulation settings that may contain EBV-positive HRS-like cells [163, 174, 175]. Caution should be exercised, therefore, when considering the diagnosis of CHL in the IDD setting; the same applies to purely extranodal CHL-like lymphoproliferations.

WHO-HAEM5 continues to list **nodular lymphocyte predominant Hodgkin lymphoma (NLPHL)** under the family of Hodgkin lymphoma; the existing terminology of NLPHL (Hodgkin lymphoma) is maintained so as not to interfere with ongoing clinical trials. However, NLPHL may be more accurately called “nodular lymphocyte predominant B-cell lymphoma” since the neoplastic cells have a functional B-cell program, and therefore this term is now considered acceptable in preparation of future definitive adoption of the new nomenclature. An important issue in NLPHL is the recognition of the different growth patterns [176] overlapping with T-cell/histiocyte-rich large B-cell lymphoma (THRLBCL) at the extreme end (Table 6) [177]. These patterns occur across all age groups. Some variant patterns (patterns C, D and E) have been associated with more aggressive clinical behaviour in retrospective analyses [177–179] and may thus reflect the natural development and progression of the tumour [180, 181]. In some cases, a clear distinction between NLPHL Pattern E and THRLBCL may not be possible since both diseases present with advanced clinical stage. Distinction is especially difficult on small biopsies, which may not be representative.

**Table 6:** Immuno-morphological growth patterns of NLPHL.

Designation	Description
Pattern A	Classic B-cell nodular
Pattern B	Serpiginous/interconnected
Pattern C	Prominent extra-nodular LP cells
Pattern D	T-cell-rich nodular
Pattern E	Diffuse THRLBCL/DLBCL-like
Pattern F	Diffuse moth-eaten, B-cell-rich

*THRLBCL* T-cell/histiocyte-rich large B-cell lymphoma.

### Plasma cell neoplasms and other diseases with paraproteins: new conditions from AESOP to TEMPI

The section on plasma cell neoplasms in WHO-HAEM5 recognizes new entities and incorporates structural modifications as a step forward from WHO HAEM4R. New conditions included are **monoclonal gammopathy of renal significance (MGRS)**, **cold agglutinin disease (CAD)**, as well as **TEMPI syndrome** (a provisional entity in WHO-HAEM4R, characterized by telangiectasias, elevated erythropoietin and erythrocytosis, monoclonal gammopathy, perinephric fluid collection, and intrapulmonary shunting) and **AESOP syndrome** (adenopathy and extensive skin patch overlying a plasmacytoma). Sections based on types of paraproteins and disease burden have been reorganised. CAD, IgM and non-IgM MGUS and MGRS are grouped as monoclonal gammopathies, and diseases with abnormal monoclonal immunoglobulin deposits are grouped together. The heavy chain diseases (HCD) are now included in the plasma cell neoplasms section.

**Cold agglutinin disease (CAD)** is an autoimmune haemolytic anemia mediated by monoclonal cold agglutinins and driven by an underlying clonal B-cell lymphoid proliferation not fulfilling criteria for a B-cell lymphoma. The annual incidence of this rare disease is estimated at 1–1.8 per million; its prevalence is four-fold higher in colder countries [182–184]. **Monoclonal gammopathy of renal significance (MGRS)** represents a plasma cell or B-cell proliferation that does not meet accepted criteria for malignancy but secretes a monoclonal immunoglobulin or immunoglobulin fragment resulting in kidney injury [185, 186]. About 1.5% of patients whose disease would otherwise be classified as MGUS have MGRS [187].

The risk stratification model for **IgM MGUS** and **non-IgM MGUS** has been updated. Presence of all 3 risk factors consisting of: (1) an abnormal serum free light chain ratio, (2) IgA or IgM type MGUS, and (3) serum M-protein value >1.5 g/dL is considered high risk with approximately 50–60% risk of progression at 20 years, whereas the risk is only 5% when none of the risk factors are present [188]. A diagnosis of **TEMPI syndrome** is primarily made on clinical and imaging investigations. The bone marrow is unremarkable in the majority of cases; a few cases show erythroid hyperplasia and a low-volume of light chain-restricted plasma cells [189, 190]. Skin biopsies of patients with **AESOP syndrome** show diffuse hyperplasia of dermal vessels associated with surrounding dermal mucin, and lymph nodes can show features mimicking Castleman disease [191, 192].

New data have emerged concerning the progression from precursor states to **plasma cell (multiple) myeloma (PCM)**, involving branching evolutionary patterns, novel mutations, biallelic hits in tumour suppressor genes, and segmental copy number changes [193]. While 1q21 gain is often an early event, translocations and additional amplifications of 1q21 emerge later during pathogenesis [194]. Staging of PCM according to the Revised International Staging System for Multiple Myeloma proposed by the International Myeloma Working Group has been adopted [195]. The important role of minimal/measurable residual disease (MRD) using next-generation flow cytometry or next-generation sequencing of immunoglobulin gene rearrangements as well as PET/CT in assessing prognosis and risk stratification in patients with PCM has been detailed [196, 197].

### T-cell and NK-cell lymphoid proliferations and lymphomas

WHO-HAEM5 has reorganized entities that were listed as mature T- and NK-cell neoplasms in WHO HAEM4R to include a broader group of entities under the heading of “T-cell and NK-cell lymphoid proliferations and lymphomas” (Table 2). Notably, included is a family/class of tumour-like lesions with T cell predominance. Precursor T-lymphoblastic neoplasms are also included under this overarching category as a separate family. The mature T-cell and NK-cell neoplasms are grouped into 9 families based on diverse concepts: cell of origin/differentiation state, clinical scenario, disease localization, and cytomorphology. While most T- or NK-cell neoplasms can be assigned to the respective T- or NK-cell lineage, they are not separated as two categories in WHO-HAEM5 because some entities comprise a spectrum of tumours of NK, T, hybrid or indeterminate phenotype, such as in extranodal NK/T-cell lymphoma, EBV+ nodal T- and NK-cell lymphoma, chronic active EBV disease and severe mosquito bite allergy. In other instances, distinction between T- and NK-cell origin may be unclear or difficult to determine.

### Tumour-like lesions with T-cell predominance: a new class of tumour-like lesions

The new family of **tumour-like lesions with T-cell predominance** in WHO-HAEM5 includes three distinct entities: indolent T-lymphoblastic proliferation (ITLP), Kikuchi-Fujimoto disease (KFD), and autoimmune lymphoproliferative syndrome (ALPS). These expansions of T cells can potentially be mistaken for lymphoma. **Indolent T-lymphoblastic proliferation (ITLP)** may occur by itself or in association with benign and neoplastic follicular dendritic cell proliferations and other malignancies. It shows clusters or confluent sheets of lymphoid cells which can range in appearance from small lymphocytes to slightly larger cells with more open chromatin (morphologically consistent with thymocytes as seen in the normal thymus), which may be mistaken for T-lymphoblastic leukaemia/lymphoma due to TdT expression [198–205]. However, ITLP may distort, but typically does not obliterate the architecture of the involved tissues, the TdT+ cells are not as atypical as those encountered in lymphoblastic leukaemia/lymphoma, and ITLP does not show monoclonal TCR gene rearrangement. **Kikuchi-Fujimoto disease (KFD)** commonly shows large aggregates and sheets of T immunoblasts and histiocytes, accompanied by prominent apoptosis in lymph nodes, mimicking peripheral T-cell lymphoma NOS. Clues to the correct diagnosis include the typical clinical scenario of cervical lymphadenopathy in a young woman, the circumscribed and non-expansile nature of the nodal infiltrate, presence of a significant component of plasmacytoid dendritic cells (CD123+) and presence of many histiocytes that express myeloperoxidase. **Autoimmune lymphoproliferative syndrome (ALPS)**, which is associated with autoimmunity and germline or somatic pathogenetic changes in genes involved in FAS-mediated apoptosis [206], has nodal or extranodal infiltrates of CD4-, CD8- T cells, which may appear as atypical medium-sized cells with clear cytoplasm that may mimic lymphoma. The clinical setting (young patient age) and lack of destructive infiltrate may provide clues to its benign nature [207].

### Precursor T-cell neoplasms: uncertainties about NK-lymphoblastic leukaemia/lymphoma

**T-lymphoblastic leukaemia/lymphoma (T-ALL)** are precursor T-cell neoplasms, comprising T-lymphoblastic leukaemia/lymphoma NOS and early T-precursor lymphoblastic leukaemia/lymphoma, as in WHO-HAEM4R. The latter shows a gene expression signature corresponding to that of earlier stages of normal precursor T cells as compared with the former entity, and shows a unique immunophenotype that includes expression of stem cell and/or myeloid markers. Despite significant advances in our understanding of the genetic background of T-ALL [208], there is as yet not sufficient evidence to establish genetically defined types of T-ALL with clinical relevance.

NK-lymphoblastic leukaemia/lymphoma, considered a provisional entity in WHO-HAEM4R, is not separately listed in WHO-HAEM5 because of lack of clear-cut and reliable diagnostic criteria, lack of published information on expression on NK-cell-associated antigens such as CD94 and CD161, and marked morphologic and immunophenotypic overlap with other entities, such as blastic plasmacytoid dendritic cell neoplasm, CD56+ T-ALL, CD56+ acute myeloid leukaemia and CD56+ acute undifferentiated leukaemia [209].

### Mature T-cell and NK-cell leukaemias: a family is growing

The family of **mature T-cell and NK-cell leukaemias** encompasses neoplastic T- and NK-cell proliferations that primarily present as leukaemic disease, including T-prolymphocytic leukaemia (T-PLL), T-large granular lymphocytic leukaemia (T-LGLL), NK-large granular lymphocytic leukaemia (NK-LGLL), adult T-cell leukaemia/lymphoma (ATLL), Sezary syndrome (SS) and aggressive NK-cell leukaemia (ANKL). Enhanced molecular understanding is considered sufficiently mature to permit incorporation of such features in the diagnostic criteria or prognostic markers of these diseases, where relevant.

**T-prolymphocytic leukaemia (T-PLL**) is a rare form of mature T-cell leukaemia with a heterogeneous clinical course. Recent efforts to standardize diagnosis, staging and treatment response [210] have led to unified diagnostic criteria, which include T lymphocytosis (>5 x 10^9^/L) with appropriate phenotype, T-cell monoclonality and the presence of genetic aberrations including structural variants with breakpoints affecting the *TCL1A* or *MTCP1* locus or expression of TCL1. There is emerging evidence of clinical and phenotypic significance of specific mutations in **T-large granular lymphocytic leukaemia (T-LGLL).** *STAT3* mutation, found preferentially in CD8+ T-LGLL and gamma/delta T-LGLL, is associated with neutropenia and poorer overall survival [211–214]. *STAT5B* mutation is over-represented in the rare CD4+ T-LGLLs (present in up to 30% of cases); it is associated with a poor prognosis in CD8+ T-LGLL, but has no prognostic impact in CD4+ T-LGLL and gamma/delta T-LGLL [214]. “Chronic lymphoproliferative disorder of NK cells” in WHO-HAEM4R has been renamed **NK-large granular lymphocytic leukaemia (NK-LGLL)**, given recent evidence that this is a monoclonal or oligoclonal expansion of NK cells that has many similarities with T-LGLL. Genetic analyses of **adult T-cell leukaemia/lymphoma (ATLL)** have revealed novel events that highlight the importance of immune evasion including *CTLA4*::*CD28* and *ICOS*::*CD28* fusions, *REL* C-terminal truncations [215, 216], recurrent alterations in *HLA-A* and *HLA-B* and structural variations disrupting the 3′-untranslated region of *CD274* (*PD-L1)* [217]. Furthermore, the frequency and pattern of somatic alterations appear to be correlated with clinical behavior. Specifically, aggressive subtypes show more genetic alterations, whereas *STAT3* mutations are more common in indolent subtypes. Based on clinical and serological features, prognostic indices of ATLL have been better defined, and a prognostically meaningful genetic classification has recently been proposed [218]. **Sezary syndrome (SS)**, while closely related to mycosis fungoides but a distinct entity, is included in this section to highlight its primary site of clinical presentation and consideration in the differential diagnosis of mature T-cell leukaemias. Comprehensive analyses of genomic signatures [219] highlight the contribution of cellular aging and UV exposure observed in SS. Genome-wide sequencing studies have provided novel insights into pathogenetic events in **aggressive NK-cell leukaemia (ANKL)**. They implicate mutations in genes of the JAK/STAT and RAS/MAPK pathways, epigenetic modifiers (*TET2, CREBBP,  KMT2D*), and immune checkpoint molecules *CD274* (*PD-L1*)/*PDCD1LG2 (PD-L2*) [220–223] in disease pathogenesis.

### Primary cutaneous T-cell lymphoid proliferations and lymphomas (CTCL): rare subtypes become entities

Primary cutaneous T-cell lymphoid proliferations and lymphomas (CTCL) comprise a dedicated family within the mature T/NK-cell neoplasms chapter in WHO-HAEM5, and include nine entities.

In WHO-HAEM4R, primary cutaneous gamma/delta T-cell lymphoma, CD8-positive aggressive epidermotropic cytotoxic T-cell lymphoma, acral CD8-positive T-cell lymphoproliferative disorder and CD4-positive small or medium T-cell lymphoproliferative disorder were grouped together under the term ‘cutaneous peripheral T-cell lymphoma, rare subtypes’, but are now each listed as separate entities in WHO-HAEM5 acknowledging their specific clinicopathological and genetic characteristics. The variants of mycosis fungoides from WHO-HAEM4R remain in place as subtypes; however, within the folliculotropic category, clinical early versus advanced stage patterns are described, and should be distinguished, to acknowledge differing clinical outcomes. There still remain rare cases that do not fit into the other known CTCL entities, and that are grouped into the newly coined entity “primary cutaneous peripheral T-cell lymphoma, NOS”, awaiting further studies to clarify their nature [224].

As there is morphologic and immunophenotypic overlap among the various forms of primary CTCL, correlation with clinical history, signs and symptoms is a key element of the diagnostic work-up. Thus, dermatological examination and clinical photographic documentation are indispensable in reaching the correct diagnosis [224, 225].

### Intestinal T-cell and NK-cell lymphoid proliferations and lymphomas: indolent NK-cell lymphoproliferative disorder as the new kid in town

**Fig. 6:**
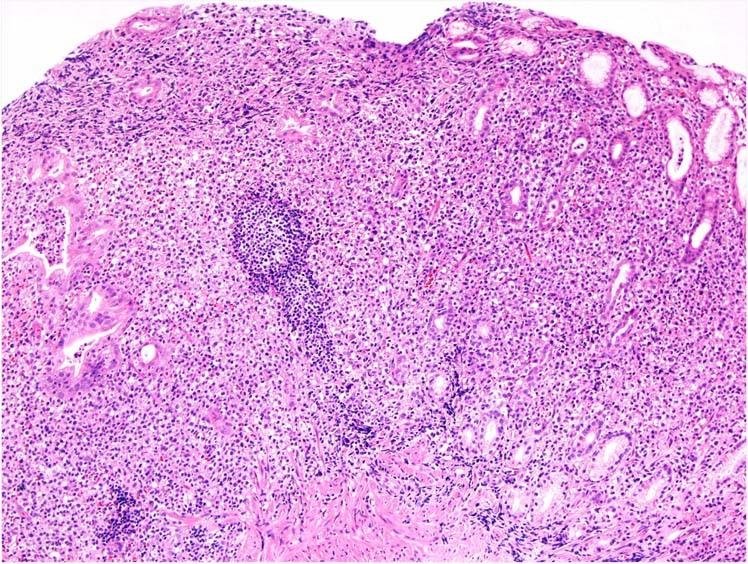
Indolent NK-cell lymphoproliferative disorder of the gastrointestinal tract involving the stomach. The gastric mucosa shows expansion of the lamina propria by an atypical lymphoid infiltrate. The tumour cells are medium-sized, often with pale-staining cytoplasm.

In WHO-HAEM5, the main changes in the classification of intestinal T-cell and NK-cell lymphomas include: new nomenclature for indolent T-cell lymphoproliferative disorder of the gastrointestinal tract, now designated “indolent T-cell lymphoma of the gastrointestinal (GI) tract”, and addition of a new entity, “indolent NK-cell lymphoproliferative disorder of the GI tract” (iNKLPD) (Table 7). For **indolent T-cell lymphoma of the gastrointetinal (GI) tract**, the change from the conservative designation of “lymphoproliferative disorder” to “lymphoma” is justified by the significant morbidity related to the tumour and the ability of the disease to disseminate, while the qualifier “indolent” remains to indicate its protracted clinical course [226–230]. There are interesting correlations between T-cell subsets and genetic changes in this neoplasm: alterations in JAK-STAT pathway genes and mutations in epigenetic modifier genes (e.g., *TET2*, *KMT2D*) preferentially occur in CD4+, CD4+/CD8+, and CD4-/CD8- subsets, with CD4+ cases sometimes displaying *STAT3*::*JAK2* fusions. In contrast, some CD8+ cases have been shown to harbor structural alterations involving the *IL2* gene [227, 230]. **Indolent NK-cell lymphoproliferative disorder of the GI tract (iNKLPD)**, (Fig. 6) formerly known as lymphomatoid gastropathy or NK-cell enteropathy and previously thought to be a reactive process, is included as an entity because of recent findings supporting its neoplastic nature. Somatic mutations in various genes have been identified, including recurrent *JAK3* mutations (K563_C565del; NP_000206). Moreover, immunophenotypic features support a role for JAK3-STAT5 pathway activation in pathogenesis [231]. Nonetheless, the disease has a benign clinical outcome: individual lesions usually spontaneously regress in a few months, although lesions may persist or new lesions may develop over a period of years. Progression to aggressive disease is not reported, justifying its designation as “lymphoproliferative disorder” [231–233]. An interesting observation is that this tumour may not be entirely confined to the GI tract, with rare cases reported to involve gallbladder, adjacent lymph nodes and the vagina [234–236]. It is most important not to misinterpret iNKLPD as extranodal NK/T-cell lymphoma, the immunophenotype of which can be largely identical with the exception of crucial differential EBV association. While the infiltrate of atypical medium-sized lymphoid cells is worrisome, the small size and superficial nature of the lesions, expansile rather than highly destructive growth and presence of paranuclear brightly eosinophilic granules may provide a clue to the diagnosis, which can be further confirmed by the lack of EBV.

**Table 7:** Comparison of different types of T and NK cell lymphoproliferative disorders and lymphomas involving the gastrointestinal tract (GIT).

	Indolent T-cell lymphoma of the GIT	Indolent NK-cell LPD of the GIT	Enteropathy-associated T-cell lymphoma	Monomorphic epitheliotropic intestinal T-cell lymphoma	Extranodal NK/T-cell lymphoma
Major clinical presentation	Abdominal symptoms	Asymptomatic or nonspecific GI symptoms	Abdominal symptoms; bowel perforation or obstruction common.	Abdominal symptoms; bowel perforation or obstruction common.	Abdominal symptoms; bowel perforation common.
Association with celiac disease	–	–	+	–	–
Clinical course	Chronic persistent or relapsing	Usually spontaneous regression, but may persist or develop new lesions	Aggressive	Aggressive	Aggressive
Commonest localization in GIT	Small bowel or colon	Stomach, small and large intestines	Small intestine	Small intestine	Small and large intestines
Depth of involvement	Superficial	Superficial	Deep	Deep	Deep
Cytomorphology	Small lymphoid cells with minimal nuclear atypia	Atypical medium-sized cells with pale cytoplasm and eosinophilic granules	Pleomorphic large or medium-sized cells, often with prominent inflammatory background	Monomorphic small to medium-sized cells	Variable cytomorphology, from small to medium-sized to large cells
Epitheliotropism	−/ focal	−/ minimal	+	+	–
Necrosis	–	–	+/−	Usually –	+
EBV association	–	–	–	–	+
Lineage	T cell, CD4+ >CD8+	NK cell	T cell, most often CD4-, CD8-	T cell, most often CD8+	NK cell (commoner) or T cell
Molecular genetics	*JAK2*::*STAT3* fusion; mutations of JAK-STAT pathway genes and epigenetic modifier genes	*JAK3* mutation	Gains of 9q34; loss of 16q12; mutations of JAK-STAT pathway genes (commonly *JAK1, STAT3*)	Gains of 9q34; loss of 16q12; mutations of *SETD2* and JAK-STAT pathway genes (commonly *JAK3, STAT5B*)	6q21-25 deletion; Mutations of JAK-STAT pathway genes, epigenetic regulators, tumor suppressor genes (*TP53, MGA*) and RNA helicase (*DDX3X)*

### Hepatosplenic T-cell lymphoma: not confined to the young

Various new findings regarding **hepatosplenic T-cell lymphoma (HSTCL)** since WHO-HAEM4R have led to refinements in WHO-HAEM5. Recent studies have shown that HSTCL is not necessarily a disease of the young; only 49% of patients were younger than 60 years of age in a recent study [237]. Of note, dyspoiesis of bone marrow elements mimicking myelodysplastic syndrome is not uncommon in marrow smears of HSTCL patients, although this does not have any clinical impact [238]. While most cases express TCRγδ (~75%) followed by TCRαβ (~25%), a small subset of cases, about 5%, are TCR-silent [239].

### Anaplastic large cell lymphoma: more genetic data in an otherwise well-defined entity

WHO-HAEM5 recognizes 3 entities within the family of **anaplastic large cell lymphomas (ALCL)**, which are mature T-cell lymphomas characterized by pleomorphic tumour cells with uniform strong expression of CD30 and often defective expression of T-lineage markers. Primary cutaneous ALCL is grouped under primary cutaneous T-cell lymphoid proliferations and lymphomas acknowledging its clinico-pathological relation to these disorders and highly favorable outcome in contrast to systemic ALK- ALCL [240, 241]. **ALK positive anaplastic large cell lymphoma (ALK+ ALCL)** has been separated from ALK-negative ALCL (ALK- ALCL) since WHO-HAEM4 based on its distinct pathogenesis [242, 243] and clinical course. **ALK- ALCL** was acknowledged as a heterogeneous entity. Recent genomic analyses have led to recognition of several genetic contexts with prognostic implications, although there are currently insufficient data to determine if these are best regarded as prognostic markers or molecular subtypes. ALK-negative ALCL bearing *TP63* rearrangements [244], loss of *TP53* [244–246] and/or overexpression of IL-2Rα [247] are associated with poor outcomes. Although initial reports suggested *DUSP22* rearrangement to be associated with a favorable 5-year overall survival comparable to ALK+ ALCL [248], more recent studies have not confirmed this association [249]. Some specific molecular alterations in ALK- ALCL have been shown to correlate with morphologic features. ALCLs with *DUSP22* rearrangement are characterized by neoplastic cells with a “doughnut cell” appearance [250] and sheet-like growth pattern with less pleomorphic cells; LEF1 expression may serve as a surrogate marker for this molecular alteration [251]. A subset of cases with Hodgkin-like morphology shows aberrant ERBB4 protein expression [252], while more anaplastic cells are seen in cases with *JAK2* rearrangement [253]. **Breast implant-associated ALCL** (BIA-ALCL) is an entity distinct from other ALK- ALCL; notably it is a usually non-invasive neoplasm arising in association with textured-surface breast implants and is associated with an excellent outcome [254]. Invasion of adjacent structures, however, worsens the prognosis. Recent studies highlight the importance of TH2 allergic inflammatory response, a role for immune-evasion via amplification of 9p24.1 and overexpression of PD-L1 in over 50% of the cases and constitutive JAK-STAT activation by somatic mutations of *STAT3, STAT5B, JAK1 and JAK2* and loss-of function mutations of *SOCS1* and *SOCS3* [255–261].

### Nodal T-follicular helper cell lymphomas: New nomenclature to unite family members

This family includes three entities of nodal T-cell lymphomas that bear the phenotype and gene expression signatures of T-follicular helper (TFH) cells [262, 263]. While the conceptual basis for the recognition of these entities is consistent with that proposed in WHO-HAEM4R, a common family terminology of nodal T-follicular helper cell lymphomas (nTFHLs) is introduced in WHO-HAEM5, with previously recognized diseases now regarded as entities within this family. Accordingly, diseases previously referred to as “angioimmunoblastic T-cell lymphoma”, “follicular T-cell lymphoma” and “peripheral T cell lymphoma with TFH phenotype” are renamed nTFHL angioimmunoblastic-type (nTFHL-AI), nTFHL follicular-type (nTFHL-F) and nTFHL not otherwise specified (nTFHL-NOS), respectively. This is to recognize their significant clinical and immunophenotypic overlap and plasticity [264, 265], as well as similar TFH gene expression signature and mutation profiles. Research in the coming years may provide data to further define the boundaries in biology between these entities or rather refute such differences. The current classification provides a platform for such studies.

**Fig. 7:**
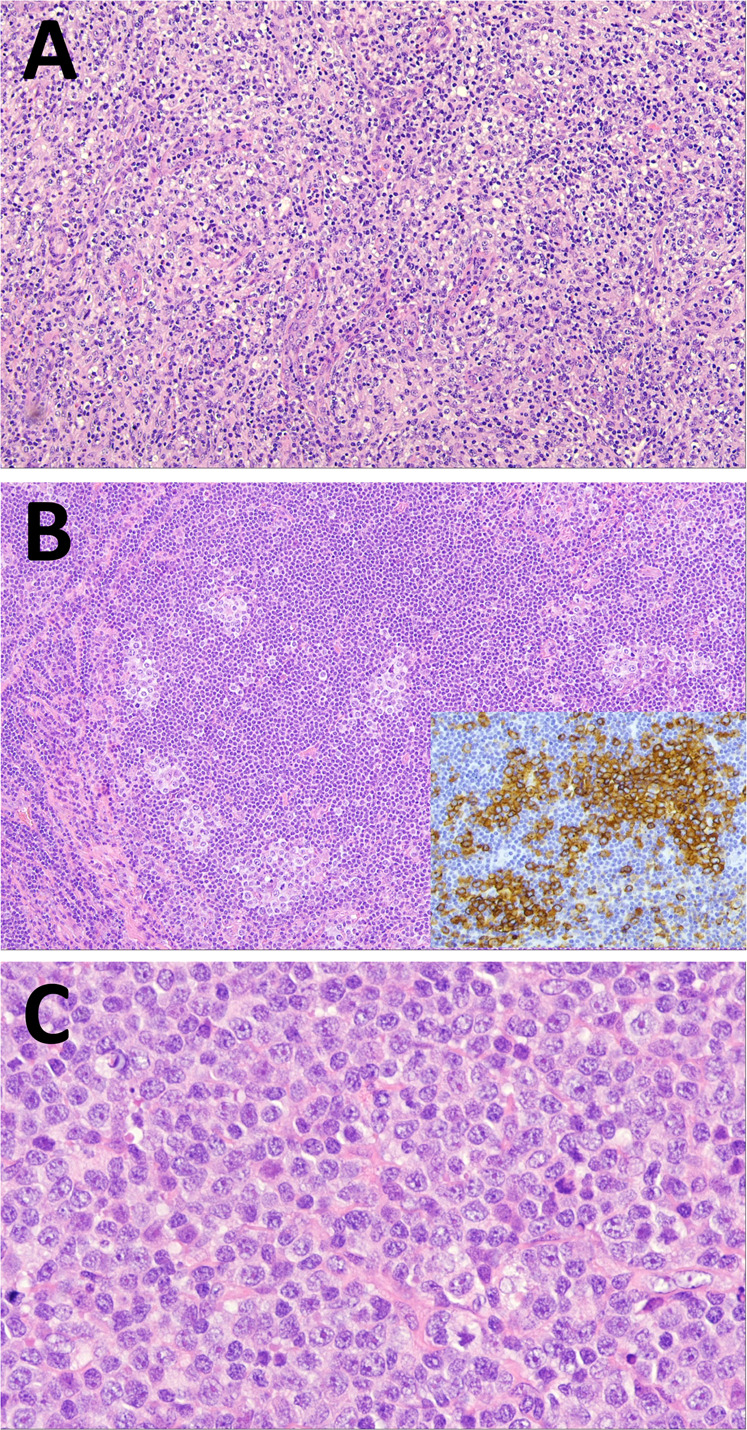
Nodal TFH-cell lymphoma (nTFHL). **A** Nodal TFH-cell lymphoma, angioimmunoblastic-type (nTFHL-AI). The normal architecture of the lymph node is effaced. There is a diffuse infiltrate of medium-sized, slightly atypical lymphocytes, sometimes with clear cytoplasm. One of the hallmarks of the disease is the proliferation of arborizing post-capillary vessels consistent with high endothelial venules. **B** Nodal TFH-cell lymphoma, follicular-type (nTFHL-F). In this example, progressive transformation of germinal centre-like nTFHL-F, clusters of atypical lymphoid cells with pale cytoplasm are embedded in a background of small lymphocytes of mantle zone type. The inset shows strong expression of PD1 in the tumour cells. **C** Nodal TFH-cell lymphoma, not otherwise specified. This tumour is composed of a sheet-like proliferation of medium-sized to large neoplastic cells.

**Nodal T-follicular helper cell lymphoma, angioimmunoblastic-type (nTFHL-AI)** is the prototype with well-defined clinical, morphologic (Fig. 7), immunophenotypic and characteristic mutational profiles. Genetically, nTFHL-AI is characterized by a stepwise acquisition of somatic changes with *TET2* and *DNMT3A* mutations occurring early in haematopoietic stem cells, while *RHOA* and *IDH2* mutations are also present in the neoplastic TFH cell population. In contrast, **nTFHL-F** and **nTFHL-NOS** (Fig. 7) represent less well-studied nodal lymphomas, which also express TFH markers such as PD1, ICOS, CXCL13, CD10, and BCL6 [266–277] and show mutation profiles similar to those of nTFHL-AI [265, 266, 278–280].

Although the individual entities are defined predominantly by histopathological features, there is considerable morphologic overlap and inter-observer variability. nTFHL-NOS is the recommended term for CD4+ lymphomas with TFH phenotype but that do not meet criteria for nTFHL-AI or nTFHL-F. The generic term nTFHL rather than nTFHL-NOS is recommended when interpreting core biopsies to prevent misclassification due to inadequate sampling. The TFH phenotype is defined as the presence of at least two TFH markers in addition to CD4. Further studies are required to determine if this definition is sufficiently robust in differentiating nTFHL-NOS from PTCL, NOS, as most cases of the former often express the less specific TFH markers such as PD1 and ICOS. In essence, the diagnosis may be challenging with many pitfalls. An integrated approach is recommended, at the very minimum requiring correlation of clinical, morphologic and immunophenotypic features, with input from genomics for clonality and mutational profiles in difficult cases.

### Other peripheral T-cell and NK-cell lymphomas: nodal EBV+ T- and NK-cell lymphoma counterpart of extranodal NK/T-cell lymphoma

In WHO-HAEM5, **peripheral T-cell lymphoma NOS (PTCL-NOS)** remains a heterogeneous category and a diagnosis of exclusion, with a differential diagnosis that in particular includes nodal T-follicular helper cell lymphomas, among others. Two possible biological variants of PTCL-NOS, PTCL-TBX21 and PTCL-GATA3, have been characterized by the transcriptional program of T-helper-1 and T-helper-2 cells, respectively [281]. While PTCL-GATA3 has a uniform molecular genetic profile, PTCL-TBX21 is heterogeneous and may include a subgroup with a cytotoxic gene expression program and aggressive behavior [281, 282]. The current status of knowledge on the genetic landscape, clinico-pathological context and prognostic implications of these possible biological variants of PTCL-NOS are deemed, however, insufficient to justify a formal status as “subtype” [283]. **Extranodal NK/T-cell lymphoma (ENKTL)** will have the qualifier “nasal-type” dropped from its name in WHO-HAEM5 in accordance with the recognized presentation of this disease at various extranodal sites. The introduction of L-asparaginase-based chemotherapy in combination with radiotherapy has led to markedly improved outcomes for this lymphoma [284]. Immune checkpoint inhibitor therapy has recently shown great promise for relapsed or refractory disease [285–287], in keeping with the finding that immune evasion is a critical pathway for ENKTL cell survival [288, 289]. **Intravascular NK/T-cell lymphoma** was considered a form of ENKTL in WHO-HAEM4R [290–295]. This highly aggressive lymphoma is often, but not invariably, EBV positive, does not present with mass lesions and shows a predilection for skin and central nervous system. Since its nosological nature is still unclear, it is now described under aggressive NK-cell leukaemia rather than extranodal NK/T-cell lymphomas, pending further studies to determine where it fits best.

**Fig. 8:**
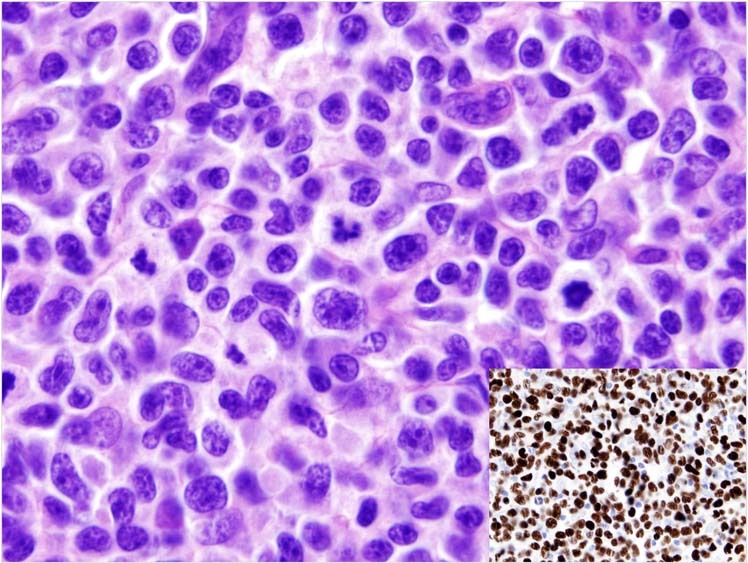
Nodal EBV-positive T- and NK-cell lymphoma. This lymphoma shows a diffuse infiltrate of relatively monotonous, medium-sized to large cells, sometimes reminiscent of centroblasts. Inset: in-situ hybridization for EBERs identifies EBV infection in virtually all tumour cells.

**Nodal EBV-positive T and NK-cell lymphoma**, which occurs mostly in East Asians [296–300], is now recognized as a distinct entity in WHO-HAEM5; previously it was subsumed as a subtype under the entity of PTCL-NOS. Patients typically present with lymphadenopathy with or without extranodal involvement, advanced-stage disease and B symptoms; they have a dismal prognosis. Morphologically, this lymphoma often resembles diffuse large B-cell lymphoma, lacking the coagulative necrosis and angioinvasion characteristic of ENKTL (Fig. 8). It more often shows a cytotoxic T-cell than NK-cell immunophenotype; EBV is positive, by definition. The genetic landscape differs from that of ENKTL, with the most commonly mutated gene being *TET2* [300].

### EBV-positive T- and NK-cell lymphoid proliferations and lymphomas of childhood: revised terminology

EBV-associated lymphoid proliferations and lymphomas of childhood are uncommon T- and NK-cell disorders with a predilection for Asian and native American ethnic groups [301–305]; occurrence in adults is also reported [306]. This family includes **chronic active EBV disease (CAEBVD) and systemic EBV-positive T-cell lymphoma of childhood**. CAEBVD is characterized by a broad clinical spectrum varying from localized and/or indolent forms (**severe mosquito bite allergy** and **hydroa vacciniforme lymphoproliferative disorder [HVLPD] classic form**), to systemic disease with fever, hepatosplenomegaly, and lymphadenopathy, with or without cutaneous manifestations (**HVLPD systemic form** and **systemic CAEBVD**).

This classification introduces several changes in terminology to reflect the morphologic overlap among different entities, such as HVLPD systemic form and systemic CAEBVD, and the need for clinicopathologic correlation in diagnosis. “Hydroa vacciniforme-like lymphoproliferative disorder” in WHO-HAEM4R has been renamed HVLPD, with identification of a classic and a systemic form. Systemic HVLPD shows persistent systemic symptoms of CAEBVD or extracutaneous disease, and should be distinguished from systemic CAEBVD without HVLPD, which is characteried by an even more aggressive clinical course [307–310]. Moreover, the usually fatal outcome in the absence of haematopoietic stem cell transplantation has led to replacement of the former terminology “chronic active EBV **infection**, systemic form” with “systemic chronic active EBV **disease**” [308, 309].

### Stroma-derived neoplasms of lymphoid tissues: some tumour types are unique to lymph node or spleen

A new category of stroma-derived neoplasms of lymphoid tissues is introduced in WHO-HAEM5 (Table 3). Mesenchymal tumours specific to lymph node (including **intranodal palisaded myofibroblastoma)** and spleen (including **littoral cell angioma, splenic hamartoma and sclerosing angiomatoid nodular transformation)** are covered, while various soft tissue tumours not specific to lymph node or spleen (such as haemangioma, lymphangiomyoma, Kaposi sarcoma and angiosarcoma) are covered in the WHO Classification of Soft Tissue and Bone Tumours (5^th^ edition, 2020). Furthermore, follicular dendritic cell and fibroblastic reticular cell neoplasms have been moved from the “histiocytic and dendritic cell neoplasms” category to this new category, because follicular dendritic cells are not derived from haematopoietic stem cells but rather are of mesenchymal origin [311–313]. Given its distinctive clinicopathologic features, **EBV-positive inflammatory follicular dendritic cell sarcoma** is delineated as an entity separate from follicular dendritic cell sarcoma [314], together with a nomenclature change from “inflammatory pseudotumour-like follicular/fibroblastic dendritic cell sarcoma”, a change first introduced in the WHO Classification of Digestive Tract Tumours (5^th^ edition, 2019) [315].

### Genetic predisposition syndromes: increasing importance of germline genetics

To acknowledge the growing number of known germline predispositions associated with haematologic neoplasms, lymphoid neoplasms occurring in the context of clinical syndromes should be separately recognized, similar to classification in other organ systems. To this end, WHO-HAEM5 introduces new chapters on genetic predisposition. With regard to lymphoid neoplasms, Ataxia telangiectasia (AT) and Nijmegen-Breakage syndrome are particularly relevant. These are linked to germline mutations in *ATM* and *NBN*, respectively. The detection of such underlying syndromes associated with germline predisposition is clinically important not only with regards to treatment planning (e.g., increased toxicities) but also surveillance of carriers and counselling of relatives. In this regard, leukaemias and lymphomas should be diagnosed using conventional criteria but should be designated as “AT-related” or “NBS-related”. Besides the separate chapter on genetic predisposition syndromes, aspects of germline predisposition including recommendations for germline testing have been systematically incorporated in individual chapters.

### Epilogue

The dramatic increase in information regarding lymphoid tumours and their molecular complexity suggests that Fyodor Dostoyevsky’s famous words [316], “*Reality is infinitely diverse (…and) Reality resists classification*” hold true for lymphoma classifications.

We are aware that any classification is arbitrary and subject to further evolution as new evidence arises. Moreover, since the development and differentiation of lymphocytes represent a continuous spectrum rather than a sequence of distinct steps, we acknowledge that any classification system breaks up a disease continuum into groups using arbitrary (and yet evidence-based) borders. Furthermore, our daily work demands the naming, and hence, the diagnosis, of discrete entities to allow for treatment decisions and for patient management. Therefore, following the principles of Linnaeus, classification also provides the basis for preserving knowledge and providing a template for future work.

We are grateful to - and stand on the shoulders of - countless individuals and research teams, who have contributed significantly to establish the foundations of the current lymphoma classification. We thank the numerous authors and contributors whose input and thoughts have created the herein outlined ‘snapshot-in-time’ of the classification. We are confident that the present proposal, albeit by definition imperfect, will provide a robust framework for future generations of scientists to continue our efforts, to further disentangle the universe of lymphoma biology, for the benefit of patient care.

## Disclaimer

The content of this article represents the personal views of the authors and does not represent the views of the authors’ employers and associated institutions. This work is intended to provide a preview and summary of content whose copyright belongs solely to the International Agency for Research on Cancer/World Health Organization. Any or all portions of the material in this work may appear in future International Agency for Research on Cancer/World Health Organization publications.
