# Correction: “The 5th edition of The World Health Organization Classification of Haematolymphoid Tumours: Lymphoid Neoplasms” Leukemia. 2022 Jul;36(7):1720–1748

**DOI:** 10.1038/s41375-023-01962-5

**Published:** 2023-07-19

**Authors:** Rita Alaggio, Catalina Amador, Ioannis Anagnostopoulos, Ayoma D. Attygalle, Iguaracyra Barreto de Oliveira Araujo, Emilio Berti, Govind Bhagat, Anita Maria Borges, Daniel Boyer, Mariarita Calaminici, Amy Chadburn, John K. C. Chan, Wah Cheuk, Wee-Joo Chng, John K. Choi, Shih-Sung Chuang, Sarah E. Coupland, Magdalena Czader, Sandeep S. Dave, Daphne de Jong, Arianna Di Napoli, Ming-Qing Du, Kojo S. Elenitoba-Johnson, Judith Ferry, Julia Geyer, Dita Gratzinger, Joan Guitart, Sumeet Gujral, Marian Harris, Christine J. Harrison, Sylvia Hartmann, Andreas Hochhaus, Patty M. Jansen, Kennosuke Karube, Werner Kempf, Joseph Khoury, Hiroshi Kimura, Wolfram Klapper, Alexandra E. Kovach, Shaji Kumar, Alexander J. Lazar, Stefano Lazzi, Lorenzo Leoncini, Nelson Leung, Vasiliki Leventaki, Xiao-Qiu Li, Megan S. Lim, Wei-Ping Liu, Abner Louissaint, Andrea Marcogliese, L. Jeffrey Medeiros, Michael Michal, Roberto N. Miranda, Christina Mitteldorf, Santiago Montes-Moreno, William Morice, Valentina Nardi, Kikkeri N. Naresh, Yasodha Natkunam, Siok-Bian Ng, Ilske Oschlies, German Ott, Marie Parrens, Melissa Pulitzer, S. Vincent Rajkumar, Andrew C. Rawstron, Karen Rech, Andreas Rosenwald, Jonathan Said, Clémentine Sarkozy, Shahin Sayed, Caner Saygin, Anna Schuh, William Sewell, Reiner Siebert, Aliyah R. Sohani, Ritsuro Suzuki, Reuben Tooze, Alexandra Traverse-Glehen, Francisco Vega, Beatrice Vergier, Ashutosh D. Wechalekar, Brent Wood, Luc Xerri, Wenbin Xiao, the International Agency for Research on Cancer/World Health Organization, Norah Olubunmi Akinola, Yassmine Akkari, Luis M. Allende, Katsuyuki Aozasa, Iguaracyra Araujo, Luca Arcaini, Kirit M. Ardeshna, Naoko Asano, Andishe Attarbaschi, Chris M. Bacon, Sharon Louise Barrans, Tracy Batchelor, Maxime Battistella, Linda B. Baughn, Amir Behdad, Sigbjørn Berentsen, Giada Bianchi, Jacob Bledsoe, Peter Borchmann, Mark Bower, Barbara Buldini, Jan Andreas Burger, Birgit Burkhardt, Ryan D. Cassaday, Giovanni Cazzaniga, Nadine Cerf-Bensussan, Ethel Cesarman, Mammen Chandy, Jennifer R. Chapman, Björn Chapuy, Xueyan Chen, Chee Leong Cheng, Carlos Chiattone, Nicholas Chiorazzi, Lucy B. Cook, Wendy A. Cooper, Gregory Philip Corboy, Andrew John Cowan, Immacolata Cozzolino, Ian A. Cree, Emanuele S. G. d’Amore, Andrew John Davies, Martina Deckert, Jan Delabie, Elizabeth G. Demicco, Vikram Deshpande, Arjan Diepstra, Daan Dierickx, Kieron Dunleavy, Barbara Eichhorst, Daisuke Ennishi, David C. Fajgenbaum, Pedro Farinha, Carlos Fernández de Larrea, Kevin E. Fisher, Jude Fitzgibbon, Melina Flanagan, Jonathan Fromm, Juan F. Garcia, William Robert Geddie, Morie Gertz, Ajay Gopal, Satish Gopal, Patricia Theresa Greipp, Alejandro Gru, Ritu Gupta, Martin-Leo Hansmann, Konnie M. Hebeda, Klaus Herfarth, Marco Herling, Olivier Hermine, Khe Hoang-Xuan, Jennelle Hodge, Shimin Hu, Yuhua Huang, Yin Pun Hung, Stephen Hunger, Hiroto Inaba, Hiroshi Inagaki, Javeed Iqbal, Kenji Ishitsuka, Noriko Iwaki, Keiji Iwatsuki, Nitin Jain, Yoon Kyung Jeon, Marshall Kadin, Sachiko Kaji, Aanchal Kakkar, Anastasios Karadimitris, Keisuke Kataoka, Seiichi Kato, Marie José Kersten, Rhett P. Ketterling, Ji Eun Kim, Christian P. Kratz, Robert Kridel, Sigurdur Kristinsson, Ralf Küppers, Isinsu Kuzu, Yok-Lam Kwong, Ann Lacasce, Laurence Lamant-Rochaix, Thierry Lamy, Ola Landgren, Siddhartha Laskar, William Bradlyn Laskin, Georg Lenz, Shaoying Li, Gan Di Li, Pei Lin, Franco Locatelli, Robert Brian Lorsbach, Izidore Lossos, Thomas P. Loughran, William R. Macon, Joseph J. Maleszewski, Pankaj Malhotra, Teresa Marafioti, Dai Maruyama, Alexander Marx, Sam M. Mbulaiteye, Veronique Meignin, Ester Mejstrikova, Pamela Michelow, Markku Miettinen, Rodney R. Miles, Hiroaki Miyoshi, Thierry Jo Molina, Manuela Mollejo, Shuji Momose, Tetsuya Mori, William G. Morice, Bertrand Nadel, Hirokazu Nagai, Motoo Nagane, Reena Nair, Naoya Nakamura, Atsuko Nakazawa, Samih Nasr, Andrew Gordon Nicholson, Alina Nicolae, Robert Shigeo Ohgami, Naoki Oishi, Timothy S. Olson, Nicolas Ortonne, Bruno Paiva, Qiang Pan-Hammarström, Mayur Parihar, Marco Paulli, Andrea Petersen, Jennifer Picarsic, Alessandro Pileri, Nicola Pimpinelli, Jose A. Plaza, Karen R. Rabin, Markus Raderer, Kanti Rai, Ulla Randen, Huilan Rao, Alistair Robson, Rosemary Rochford, Richard Rosenquist, Davide Rossi, Esther D. Rossi, Simon Rule, Grzegorz Rymkiewicz, Elena Sabattini, Vaskar Saha, Mamiko Sakata-Yanagimoto, Christian A. Sander, J. Martin Sangueza, Omar P. Sangüeza, Marco Santucci, Yasuharu Sato, Akira Satou, Kristian Theo Schafernak, Fernando Schmitt, Gianpietro Semenzato, Manju Sengar, Tait Shanafelt, Kazuyuki Shimada, Graham W. Slack, Susan Slager, Riccardo Soffietti, David A. Solomon, Kostas Stamatopoulos, Christian Steidl, Stephan Stilgenbauer, Narittee Sukswai, Kengo Takeuchi, Giovanni Tallini, Junichi Tamaru, Soo-Yong Tan, Prashant Tembhare, Enrico Tiacci, Yoshiki Tokura, Olivier Tournilhac, Steven Treon, Lorenz Truemper, Kunihiro Tsukasaki, Frits van Rhee, Abraham Varghese, Maarten H. Vermeer, Philippe Vielh, Brian Walker, Michael Wang, Huan-You Wang, Zhe Wang, Takashi Watanabe, Oliver Weigert, David Weinstock, Sean J. Whittaker, Rein Willemze, Wilhelm Woessmann, Catherine J. Wu, Motoko Yamaguchi, Hidetaka Yamamoto, Daisuke Yamashita, Shenmiao Yang, David T. Yang, Takahiko Yasuda, Wei-Hua Yin, Yoh Zen, Sha Zhao, Wei-Li Zhao

**Affiliations:** 1 Pathology Unit, Department of Laboratories, Bambino Gesu Children’s Hospital, IRCCS, Rome, Italy; 2 Department of Pathology, University of Miami, Miami, FL USA; 3 Institute of Pathology, Julius-Maximilians-Universität Würzburg, Würzburg, Germany; 4 Department of Histopathology, Royal Marsden Hospital, London, UK; 5 Department of Pathology, Federal University of Bahia (UFBA), Salvador, Brazil; 6 University of Milan, Fondazione Cà Granda, IRCCS, Ospedale Maggiore Policlinico, Milan, Italy; 7 Department of Pathology and Cell Biology, Columbia University Irving Medical Center, New York, NY USA; 8 Division of Histopathology, SL Raheja Hospital, Mumbai, India; 9 Department of Pathology, University of Michigan, Ann Arbor, MI USA; 10 Centre for Haemato-Oncology, Barts Cancer Institute, QMUL and SIHMDS Barts Health NHS Trust, London, UK; 11 Department of Pathology and Laboratory Medicine, Weill Cornell Medicine, New York, NY USA; 12 Department of Pathology, Queen Elizabeth Hospital, Kowloon, Hong Kong; 13 National University Cancer Institute, Singapore, Singapore; 14 Department of Pathology, The University of Alabama at Birmingham, Birmingham, AL USA; 15 Department of Pathology, Chi-Mei Medical Center, Tainan, Taiwan; 16 Liverpool Clinical Laboratories, Liverpool University Hospitals Foundation Trust, Liverpool, UK; 17 Department of Pathology and Laboratory Medicine, Indiana University, Indianapolis, IN USA; 18 Center for Genomic and Computational Biology and Department of Medicine, Duke University, Durham, NC USA; 19 Amsterdam UMC, Location Vrije Universiteit Amsterdam, Department of Pathology, Amsterdam, The Netherlands; 20 Department of Clinical and Molecular Medicine, Sapienza University, School of Medicine and Psychology, Sant’ Andrea Hospital, Rome, Italy; 21 Division of Cellular and Molecular Pathology, Department of Pathology, University of Cambridge, Cambridge, UK; 22 Department of Pathology and Laboratory Medicine, University of Pennsylvania, Philadelphia, PA USA; 23 Department of Pathology, Massachusetts General Hospital and Harvard Medical School, Boston, MA USA; 24 Department of Pathology, Stanford University School of Medicine, Stanford, CA USA; 25 Department of Dermatology, Northwestern University Feinberg Medical School, Chicago, IL USA; 26 Department of Pathology, Tata Memorial Hospital, Mumbai, India; 27 Department of Pathology, Boston Children’s Hospital, Boston, MA USA; 28 Translational and Clinical Research Institute, Newcastle University Centre for Cancer, Faculty of Medical Sciences, Newcastle University, Newcastle-upon-Tyne, UK; 29 Dr. Senckenberg Institute of Pathology, Goethe University Frankfurt, Frankfurt am Main, Germany; 30 Hematology/Oncology, Universitätsklinikum Jena, Jena, Germany; 31 Leiden University Medical Center, Department of Pathology, Leiden, The Netherlands; 32 Department of Pathology and Laboratory Medicine, Nagoya, Japan; 33 Kempf und Pfaltz Histologische Diagnostik Zurich, and Department of Dermatology, University Hospital Zurich, Zurich, Switzerland; 34 Department of Hematopathology, Division of Pathology and Laboratory Medicine, The University of Texas MD Anderson Cancer Center, Houston, TX USA; 35 Department of Virology, Nagoya University Graduate School of Medicine, Nagoya, Japan; 36 Department of Pathology, Hematopathology Section and Lymph Node Registry, University Hospital Schleswig-Holstein, University of Kiel, Kiel, Germany; 37 Department of Pathology and Laboratory Medicine, Children’s Hospital Los Angeles, Los Angeles, CA USA; 38 Division of Hematology, Mayo Clinic, Rochester, MN USA; 39 Departments of Pathology & Genomic Medicine, The University of Texas MD Anderson Cancer Center, Houston, TX USA; 40 Department of Medical Biotechnology, University of Siena, Siena, Italy; 41 Division of Nephrology and Hypertension, Division of Hematology, Mayo Clinic, Rochester, MN USA; 42 Department of Pathology, Medical College of Wisconsin and Children’s Wisconsin, Milwaukee, WI USA; 43 Department of Pathology, Fudan University Shanghai Cancer Center, Shanghai, PR China; 44 Department of Pathology, West-China Hospital, Sichuan University, Chengdu, PR China; 45 Department of Pathology & Immunology, Baylor College of Medicine and Texas Children’s Hospital, Houston, TX USA; 46 Department of Pathology, Charles University in Prague, Faculty of Medicine in Plzen, Plzen, Czech Republic; 47 Department of Dermatology, Venereology and Allergology, University Medical Center Göttingen, Göttingen, Germany; 48 Anatomic Pathology Department and Translational Hematopathology Lab, Valdecilla/IDIVAL University Hospital, Santander, Spain; 49 Department of Laboratory Medicine and Pathology, Mayo Clinic, Rochester, MN USA; 50 Section of Pathology, Clinical Research Division, Fred Hutchinson Cancer Center, Seattle, WA USA; 51 Department of Pathology, Yong Loo Lin School of Medicine, National University of Singapore, Singapore, Singapore; 52 Department of Clinical Pathology, Robert-Bosch-Krankenhaus, and Dr. Margarete Fischer-Bosch Institute of Clinical Pharmacology, Stuttgart, Germany; 53 Department of Pathology, Bordeaux University Hospital, Bordeaux, France; 54 Department of Pathology and Laboratory Medicine, Memorial Sloan Kettering Cancer Center, New York, NY USA; 55 Division of Hematology, Mayo Clinic, Rochester, Minnesota, Rochester, MN USA; 56 HMDS, Leeds Cancer Centre, Leeds Teaching Hospitals NHS Trust, Leeds, UK; 57 Department of Pathology and Laboratory Medicine, University of California Los Angeles, Los Angeles, CA USA; 58 Institut Curie, Hematology Department, Saint Cloud, France; 59 Department of Pathology—Aga Khan University Hospital—Nairobi, Nairobi, Kenya; 60 Section of Hematology/Oncology, University of Chicago, Chicago, IL USA; 61 Department of Oncology, University of Oxford, Oxford, UK; 62 Immunology Division, Garvan Institute of Medical Research, Sydney, NSW Australia; 63 Institute of Human Genetics, Ulm University and Ulm University Medical Center, Ulm, Germany; 64 Department of Hematology & Oncology, Shimane University School of Medicine, Shimane, Japan; 65 Division of Haematology and Immunology, Leeds Institute of Medical Research, University of Leeds, Leeds, UK; 66 Hospices Civils de Lyon/Department of Pathology/Université Lyon 1/Centre International de Recherche en Infectiologie (CIRI) INSERM U1111—CNRS UMR5308, Lyon, France; 67 Department of Pathology, Hopital Haut-Lévêque, CHU Bordeaux, Pessac, France; 68 National Amyloidosis Centre, University College London, London, UK; 69 Department of Pathology, Institut Paoli-Calmettes and Aix-Marseille University, Marseille, France; 70 Haematology and Immunology, Obafemi Awolowo University, Ile Ife, Nigeria; 71 Cytogenetics and Molecular Pathology, Legacy Health, Portland, OR USA; 72 Immunology Department, Hospital 12 de Octubre, imas12, Madrid, Spain; 73 Department of Pathology, Osaka University Graduate School of Medicine, Suita, Osaka Japan; 74 Pathology and Forensic Medicine, Federal University of Bahia, Salvador, BA Brazil; 75 Fondazione IRCCS Policlinico San Matteo and Departement of Molecular Medicine, University of Pavia, University of Pavia, Pavia, Italy; 76 Cancer Division, University College London Hospitals, London, UK; 77 Molecular Diagnostics, Nagano Prefectural Shinshu Medical Center, Suzaka, Japan; 78 Pediatric Hematology and Oncology, St. Anna Children’s Hospital, Vienna, Austria; 79 Department of Neurology, Brigham and Women’s Hospital, Boston, MA USA; 80 Pathology Department, Hôpital Saint Louis, AP-HP Université Paris, Paris, France; 81 Department of Pathology, Northwestern University, Chicago, IL USA; 82 Department of Research and Innovation, Helse Fonna Hospital Trust, Haugesund, Norway; 83 Division of Hematology, Department of Medicine, Brigham and Women’s Hospital, Boston, MA USA; 84 1st Dept of Internal Medicine, University Hospital of Cologne, Cologne, Germany; 85 National Centre for HIV Malignancy, Chelsea & Westminster Hospital, London, UK; 86 Maternal and Child’s Health Department, University of Padova, Padova, Italy; 87 Department of Leukemia, The University of Texas MD Anderson Cancer Center, Houston, TX USA; 88 Pediatric Hematology, Oncology and BMT, University Hospital Muenster, Muenster, Germany; 89 Department of Medicine, University of Washington School of Medicine, Seattle, WA USA; 90 Pediatrics and Medical Genetics, Univ Milan Bicocca—Centro Ricerca Tettamanti, Barzano, Italy; 91 Laboratory Intestinal Immunity, Institut Imagine-Inserm U1183, Université de Paris, Paris, France; 92 Clinical Hematology & Medical Oncology, Tata Medical Center, Kolkata, India; 93 Pathology and Laboratory Medicine, University of Miami, Miami, FL USA; 94 Hematology, Oncology and Tumor Immunology, Charité, University Medical Center Berlin, Berlin, Germany; 95 Laboratory Medicine and Pathology, University of Washington, Seattle, WA USA; 96 Anatomical Pathology, Singapore General Hospital, Singapore, Singapore; 97 Hematology and Oncology, Santa Casa Midical School, Sao Paulo, Brazil; 98 Karches Center for Oncology Research, The Feinstein Institutes for Medical Research, Manhasset, NY USA; 99 Department of Haematology, Imperial College Healthcare NHS Trust, London, UK; 100 Tissue Pathology and Diagnostic Oncology, Royal Prince Alfred Hospital, NSW Health Pathology, Camperdown, NSW Australia; 101 Department of Haematology, Pathology Queensland, Herston, QLD Australia; 102 Pathology Unit, Department of Mental and Physical Health and Preventive Medicine, University of Campania “L. Vanvitelli”, Naples, Italy; 103 Evidence Synthesis and Classification Branch, International Agency for Research on Cancer, Lyon, France; 104 UOC di Anatomia Patologica, Ospedale San Bortolo, Vicenza, Italy; 105 Cancer Sciences Unit, University of Southampton, Southampton, UK; 106 Department of Neuropathology, University Hospital of Cologne, Cologne, Germany; 107 Laboratory Medicine Program, University Health Network and University of Toronto, Toronto, ON Canada; 108 Pathology and Laboratory Medicine, University Medical Center Groningen, Groningen, The Netherlands; 109 Department of Hematology, University Hospitals Leuven, Leuven, Belgium; 110 Hematology and Oncology, Lombardi Cancer Center, Georgetown University, Washington, DC USA; 111 Department I for Internal Medicine and Center for Integrated Oncology Aachen, Bonn, Cologne, Duesseldorf, University of Cologne, Cologne, Germany; 112 Department of Hematology and Oncology, Okayama University Hospital, Okayama, Japan; 113 Department of Medicine, University of Pennsylvania, Philadelphia, PA USA; 114 Department of Pathology, BC Cancer Agency, Vancouver, BC Canada; 115 Amyloidosis and Myeloma Unit, Department of Hematology, Hospital Clínic of Barcelona, IDIBAPS, University of Barcelona, Barcelona, Spain; 116 Genomics and Computational Biology, Queen Mary University of London, London, UK; 117 Department of Pathology, West Virginia University School of Medicine, Morgantown, WV USA; 118 Department of Pathology, MD Anderson Cancer Center Madrid, Madrid, Spain; 119 Center for Global Health, National Cancer Institute, Rockville, MD USA; 120 Pathology & Dermatology, University of Virginia, Charlottesville, VA USA; 121 Laboratory Oncology, All India Institute of Medical Sciences, Delhi, New Delhi, India; 122 Consultation Center for Hematopathology, Institute of Pathology and Molecular Pathology, Wuppertal, Germany; 123 Department of Pathology, The Radboud University Medical Center, Nijmegen, The Netherlands; 124 Radiation Oncology, University Hospital Heidelberg, Heidelberg, Germany; 125 Hematology, Cellular Therapy, Hemostaseology, University of Leipzig, Leipzig, Germany; 126 Hematology and Laboratory of Physiopathology and Treatment of Hematological Disorders, Necker Hospital, APHP, INSERM U1163, Imagine, Paris University, Paris, France; 127 Neuro-oncology, Hôpital Universitaire Pitié Salpêtrière, Paris, France; 128 Department of Medical and Molecular Genetics, Indiana University School of Medicine, Indianapolis, IN USA; 129 Department of Pathology, Sun Yat-sen University Cancer Center, Guangzhou, PR China; 130 Division of Oncology, Department of Pediatrics, Children’s Hospital of Philadelphia, Philadelphia, PA USA; 131 Department of Oncology, St. Jude Children’s Research Hospital, Memphis, TN USA; 132 Department of Pathology and Molecular Diagnostics, Nagoya City University, Nagoya, Japan; 133 Pathology and Microbiology, University of Nebraska Medical Center, Omaha, NE USA; 134 Department of Hematology and Rheumatology, Kagoshima University, Kagoshima, Japan; 135 Department of Hematology, National Cancer Center Hospital, Tsukiji, Chuo-ku, Tokyo, Japan; 136 Departments of Dermatology, Okayama University Graduate School of Medicine, Dentistry and Pharmaceutical Sciences, Okayama, Japan; 137 Department of Pathology, Seoul National University College of Medicine, Seoul, South Korea; 138 Department of Pathology and Laboratory Medicine, Brown University Alpert School of Medicine, Providence, RI USA; 139 Department of Pathology, Japanese Red Cross Narita Hospital, Narita, Chiba, Japan; 140 Centre for Haematology, Department of Immunology and Inflammation, Imperial College London, London, UK; 141 Division of Hematology, Department of Medicine, Keio University School of Medicine, Tokyo, Japan; 142 Department of Pathology and Molecular Diagnostics, Aichi Cancer Center Hospital, Nagoya, Japan; 143 Department of Hematology, Amsterdam University Medical Centers, Amsterdam, The Netherlands; 144 Department of Pathology, Seoul National University SNU SMG Boramae Hospital, Seoul, South Korea; 145 Department of Pediatric Hematology and Oncology, Hannover Medical School, Hannover, Germany; 146 Division of Medical Oncology and Hematology, Princess Margaret Cancer Centre—UHN, Toronto, ON Canada; 147 Faculty of Medicine, University of Iceland, Reykjavik, Iceland; 148 Institute of Cell Biology (Cancer Research), University of Duisburg-Essen, Essen, Germany; 149 Department of Pathology, University of Ankara, School of Medicine, Ankara, Turkey; 150 Department of Medicine, University of Hong Kong, Queen Mary Hospital, Hong Kong, Hong Kong; 151 Medical Oncology, Dana Farber Cancer Institute, Boston, MA USA; 152 Departement of Pathology, Institut Universitaire du cancer Toulouse Oncopole, Toulouse, France; 153 Department of Hematology, Rennes University Hospital, Rennes, France; 154 Department of Medicine, Sylvester Comprehensive Cancer Center, University of Miami, Miami, FL USA; 155 Radiation Oncology, Tata Memorial Centre, Mumbai, India; 156 Department of Pathology, Yale School of Medicine, New Haven, CT USA; 157 Department of Hematology, Oncology and Pneumology, University Hospital Münster, Münster, Germany; 158 Pediatric Hematology/Oncology, Cell and Gene Therapy, Bambino Gesù Children’s Hospital, Rome, Italy; 159 Division of Pathology, Cincinnati Children’s Hospital Medical Center, University of Cincinnati College of Medicine, Cincinnati, OH USA; 160 Hematology/Oncology, University of Virginia, Charlottesville, VA USA; 161 Clinical Hematology and Medical Oncology, Postgraduate Institute of Medical Education and Research, Chandigarh, India; 162 Department of Cellular Pathology, University College Hospital London, London, UK; 163 Hematology Oncology, Cancer Institute Hospital of Japanese Foundation of Cancer Research, Tokyo, Japan; 164 Department of Pathology, University Medical Centre Mannheim, Mannheim, Germany; 165 Infections and Immunoepidemiology Branch, DCEG, National Cancer Institute, Rockville, MD USA; 166 Department of Pediatric Hematology and Oncology, 2nd Faculty of Medicine, University Hospital Motol, Prague, Czech Republic; 167 Department of Anatomical Pathology, University of the Witwatersrand and National Health Laboratory Service, Johannesburg, South Africa; 168 Laboratory of Pathology, National Cancer Institute/NIH, Bethesda, MD USA; 169 Department of Pathology, University of Utah, Salt Lake City, UT USA; 170 Department of Pathology, Kurume University, School of Medicine, Kurume, Japan; 171 Department of Pathology, AP-HP, Hôpital Necker-Enfants Malades and Robert Debré, Université de Paris, Paris, France; 172 Department of Pathology, Hospital Universitario de Toledo, Toledo, Spain; 173 Department of Pathology, Saitama Medical University, Saitama Medical Center, Kawagoe, Japan; 174 Department of Pediatrics, St. Marianna University School of Medicine, Kawasaki, Kanagawa Japan; 175 Centre d’Immunologie de Marseille-Luminy, Inserm CNRS AMU, Marseille, France; 176 Clinical Research Center, National Hospital Organization Nagoya Medical Center, Nagoya, Japan; 177 Department of Neurosurgery, Kyorin University Faculty of Medicine, Tokyo, Japan; 178 Department of Pathology, Tokai University School of Medicine, Isehara, Japan; 179 Clinical Research, Saitama Children’s Medical Center, Saitama, Japan; 180 Department of Histopathology, Royal Brompton & Harefield NHS Foundation Trust, London, UK; 181 Department of Pathology, Hautepierre, University Hospital Strasbourg, Strasbourg, France; 182 Department of Pathology, University of California San Francisco, San Francisco, CA USA; 183 Department of Pathology, University of Yamanashi, Chuo, Yamanashi, Japan; 184 Department of Pathology, AP-HP, Hopitaux Universitaires Henri-Mondor, Creteil, France; 185 Hemato-Oncology, Clinica Universidad de Navarra, Pamplona, Spain; 186 Department of Biosciences and Nutrition, Karolinska Institutet, Huddinge, Sweden; 187 Lab Haematology/Cytogenetics & Molecular Genetics, Tata Medical Center, Kolkata, India; 188 Pathology Unit, Department of Molecular Medicine, University of Pavia, Pavia, Italy; 189 Pediatric Development and Rehab, Genetics Division, Legacy Health/Randall Children’s Hospital and Washington State University Elson S. Floyd College of Medicine, Portland, OR USA; 190 Department of Pathology, Cincinnati Childrens Hospital Medical Center, Cincinnati, OH USA; 191 Experimental, Diagnostic and Specialty Medicine Department, Dermatology Unit, Bologna, Italy; 192 Health Sciences, University of Florence Medical School, Florence, Italy; 193 Pathology and Dermatology, The Ohio State University Medical Center, Columbus, OH USA; 194 Baylor College of Medicine, Texas Children’s Cancer Center, Houston, TX USA; 195 Internal Medicine I, Division of Oncology, Medical University of Vienna, Vienna, Austria; 196 Department of Medicine, Northwell Health Cancer Institute, New Hyde Park, NY USA; 197 Department of Pathology, Akershus University Hospital /University of Oslo, Lørenskog, Norway; 198 Departamento de Diagnostico Laboratorial, IPOLFG—Servico de Anatomia Patologica, Lisboa, Portugal; 199 Immunology and Microbiology, University of Colorado, Anschutz Medical Campus, Aurora, CO USA; 200 Department of Molecular Medicine and Surgery, Karolinska Institutet, Stockholm, Sweden; 201 Department of Hematology, Oncology Institute of Southern Switzerland, Bellinzona, Switzerland; 202 Department of Anatomic Pathology and Histology, Fondazione Policlinico Universitario Agostino Gemelli-IRCCS, Rome, Italy; 203 Department of Haematology, Plymouth University Medical School, Plymouth, UK; 204 Department of Pathology and Laboratory Diagnostics, Maria Sklodowska-Curie National Research Institute of Oncology, Warsaw, Poland; 205 Haematopathology, IRCCS Azienda Ospedaliero-Universitaria, Bologna, Italy; 206 Paediatric Haematology and Oncology, Tata Translational Cancer Research Centre, Kolkata, India; 207 Department of Hematology, University of Tsukuba, Ibaraki, Japan; 208 Department of Dermatology, Asklepios Klinik St. Georg, Hamburg, Germany; 209 Pathology and Dermatology, Hospital Obrero Nro 1 CNS, Hospital General, La Paz, Spain; 210 Dept of Pathology and Dermatology, Wake Forest University, School of Medicine Medical Center Boulevard, Winston-Salem, NC USA; 211 Department of Health Sciences, Division of Histopathology and Molecular Diagnostics, University of Florence School of Human Health Sciences, Firenze, Italy; 212 Department of Pathology, Okayama University Graduate School of Health Sciences, Okayama, Japan; 213 Pathology and Laboratory Medicine, Phoenix Children’s Hospital, Phoenix, AZ USA; 214 Molecular Pathology Unit, Medical Faculty, Porto University, Porto, Portugal; 215 Department of Hematology, Veneto Institute of Molecular Medicine, University of Padua, Padova, Italy; 216 Department of Medicine, Division of Hematology, Stanford University, Palo Alto, CA USA; 217 Department of Hematology and Oncology, Nagoya University Graduate School of Medicine, Nagoya, Japan; 218 Medicine and Quality Health Sciences, Mayo Clinic, Rochester, MN USA; 219 Department of Neuro-Oncology, University of Turin and City of Health and Science Hospital, Turin, Italy; 220 Centre for Research and Technology Hellas, Institute of Applied Biosciences, Thessaloniki, Greece; 221 Pathology and Laboratory Medicine, University of British Columbia, Vancouver, BC Canada; 222 Comprehensive Cancer Center Ulm (CCCU), Ulm University, Ulm, Germany; 223 Department of Pathology, Chulalongkorn University, Bangkok, Thailand; 224 Division of Pathology, Cancer Institute, Japanese Foundation for Cancer Research, Tokyo, Japan; 225 Department of Pathology, Policlinico S.Orsola-Malpighi, Bologna, Italy; 226 Pathology, Biomedical and Sciences, Saitama Medical Center, Saitama Medical University, Kawagoe, Japan; 227 Hematopathology Laboratory, Advanced Centre for Treatment, Research and Education in Cancer, Tata Memorial Centre, Navi Mumbai, India; 228 Department of Medicine and Surgery, Institute of Hematology and Center for Hemato-Oncology Research, Perugia, Italy; 229 Allergic Disease Research Center, Chutoen General Medical Center, Kakegawa, Japan; 230 Hematology and Cell Therapy, CHU de Clermont-Ferrand, Clermont-Ferrand, France; 231 Department of Medicine, Harvard Medical School, Boston, MA USA; 232 Hematology and Medical Oncology, Universitätsmedizin Göttingen, Göttingen, Germany; 233 Department of Hematology, International Medical Center, Saitama Medical University, Hidaka, Japan; 234 Myeloma Center, UAMS Winthrop P. Rockefeller Cancer Institute, Little Rock, AR USA; 235 Department of Haematology, Little Flower Hospital and Research Centre, Angamaly, India; 236 Department of Dermatology, Leiden University Medical Center, Leiden, The Netherlands; 237 Department of Pathology, Medipath and American Hospital of Paris, Paris, France; 238 Division of Hematology Oncology, Indiana University, Indianapolis, IN USA; 239 Lymphoma and Myeloma, University of Texas MD Anderson Cancer Center, Houston, TX USA; 240 Department of Pathology, University of California San Diego, San Diego, CA USA; 241 Department of Pathology, Fourth Military Medical University, Xi’an, PR China; 242 Personalized Cancer Immunotherapy, Mie University Graduate School of Medicine, Tsu, Japan; 243 Medical Department III, Ludwig-Maximilians-University (LMU) Hospital, Munich, Germany; 244 Medical Oncology, Dana-Farber Cancer Institute and Havard Medical School, Boston, MA USA; 245 School of Basic and Medical Biosciences KCL, St. John’s Institute of Dermatology, London, UK; 246 Pediatric Hematology and Oncology, University Medical Center Hamburg-Eppendorf, Hamburg, Germany; 247 Department of Hematological Malignancies, Mie University Graduate School of Medicine, Tsu, Japan; 248 Anatomic Pathology, Kyushu University, Fukuoka, Japan; 249 Department of Pathology, Kobe City Medical Center General Hospital, Kobe, Japan; 250 Department of Hematology, Peking University Peoples’ Hospital, Peking Institute of Hematology, Beijing, PR China; 251 Department of Pathology and Laboratory Medicine, University of Wisconsin, Madison, WI USA; 252 Advanced Diagnosis, Nagoya Medical Center, Nagoya, Japan; 253 Department of Pathology, Peking University Shenzhen Hospital, Shenzhen, PR China; 254 Institute of Liver Studies, King’s College Hospital, London, UK; 255 National Center of Translational Medicine, Institute of Hematology, Shanghai, PR China

Correction to: *Leukemia* 10.1038/s41375-022-01620-2, published online 22 June 2022

At the time of original publication, there was an incomplete listing of contributing authors and their institutions. This revision corrects those omissions.

1. Dr Ritsuro Suzuki, Department of Hematology & Oncology, Shimane University School of Medicine, Shimane, Japan, was regrettably omitted from the main author list;
2. Dr Arianna Di Napoli, Department of Clinical and Molecular Medicine, Sapienza University, School of Medicine and Psychology, Sant’Andrea Hospital, Rome, Italy, was incorrectly included as a contributor in the author contribution list, and instead should be a coauthor in the main author list;
3. Dr Luis M. Allende, a contributor in the author contribution list, was assigned with an incorrect affiliation, and the correct affiliation is 70 Immunology Department, Hospital 12 de Octubre, imas12, Madrid, Spain;
4. Dr Aanchal Kakkar, a contributor in the author contribution list, was assigned with an incorrect affiliation, and the correct affiliation is Pathology, All India Institute of Medical Sciences, New Delhi, India.

The above errors have been corrected, and the corrected list of the authors and contributors and their affiliations are as follows.

Rita Alaggio^1^, Catalina Amador^2^, Ioannis Anagnostopoulos^3^, Ayoma D. Attygalle^4^, Iguaracyra Barreto de Oliveira Araujo^5^, Emilio Berti^6^, Govind Bhagat^7^, Anita Maria Borges^8^, Daniel Boyer^9^, Mariarita Calaminici^10^, Amy Chadburn^11^, John K.C. Chan^12^, Wah Cheuk^12^, Wee-Joo Chng^13^, John K. Choi^14^, Shih-Sung Chuang^15^, Sarah E. Coupland^16^, Magdalena Czader^17^, Sandeep S. Dave^18^, Daphne de Jong^19^, Arianna Di Napoli,^20^ Ming-Qing Du^21*^, Kojo S. Elenitoba-Johnson^22^, Judith Ferry^23*^, Julia Geyer^11^, Dita Gratzinger^24^, Joan Guitart^25^, Sumeet Gujral^26^, Marian Harris^27^, Christine J. Harrison^28^, Sylvia Hartmann^29^, Andreas Hochhaus^30^, Patty M. Jansen^31^, Kennosuke Karube^32^, Werner Kempf^33^, Joseph Khoury^34^, Hiroshi Kimura^35^, Wolfram Klapper^36^, Alexandra E. Kovach^37^, Shaji Kumar^38^, Alexander J. Lazar^39^, Stefano Lazzi^40^, Lorenzo Leoncini^40^, Nelson Leung^41^, Vasiliki Leventaki^42^, Xiao-Qiu Li^43^, Megan S. Lim^22^, Wei-Ping Liu^44^, Abner Louissaint Jr.^23^, Andrea Marcogliese^45^, L. Jeffrey Medeiros^34^, Michael Michal^46^, Roberto N. Miranda^34^, Christina Mitteldorf^47^, Santiago Montes-Moreno^48^, William Morice^49^, Valentina Nardi^23^, Kikkeri N. Naresh^50^, Yasodha Natkunam^24^, Siok-Bian Ng^51^, Ilske Oschlies^36^, German Ott^52*^, Marie Parrens^53^, Melissa Pulitzer^54^, S. Vincent Rajkumar^55^, Andrew C. Rawstron^56^, Karen Rech^49^, Andreas Rosenwald^3^, Jonathan Said^57^, Clémentine Sarkozy^58^, Shahin Sayed^59^, Caner Saygin^60^, Anna Schuh^61^, William Sewell^62^, Reiner Siebert^63*^, Aliyah R. Sohani^23^, Ritsuro Suzuki^64^, Reuben Tooze^65^, Alexandra Traverse-Glehen^66^, Francisco Vega^34^, Beatrice Vergier^67^, Ashutosh D. Wechalekar^68^, Brent Wood^37^, Luc Xerri^69^, Wenbin Xiao^54^

^1^Pathology Unit, Department of Laboratories, Bambino Gesu Children’s Hospital, IRCCS, Rome, Italy;

^2^Department of Pathology, University of Miami, Miami, FL, USA;

^3^Institute of Pathology, Julius-Maximilians-Universität Würzburg, Würzburg, Germany;

^4^Department of Histopathology, Royal Marsden Hospital, London, UK;

^5^Department of Pathology, Federal University of Bahia (UFBA), Salvador, Brazil;

^6^University of Milan, Fondazione Cà Granda, IRCCS, Ospedale Maggiore Policlinico, Milan, Italy;

^7^Department of Pathology and Cell Biology, Columbia University Irving Medical Center, New York, NY, USA;

^8^Division of Histopathology, SL Raheja Hospital, Mumbai, India;

^9^Department of Pathology, University of Michigan, Ann Arbor, MI, USA;

^10^Centre for Haemato-Oncology, Barts Cancer institute, QMUL and SIHMDS Barts Health NHS Trust, London, UK;

^11^Department of Pathology and Laboratory Medicine, Weill Cornell Medicine, New York, NY, USA;

^12^Department of Pathology, Queen Elizabeth Hospital, Kowloon, Hong Kong;

^13^National University Cancer Institute, Singapore, Singapore;

^14^Department of Pathology, The University of Alabama at Birmingham, Birmingham, AL, USA

^15^Department of Pathology, Chi-Mei Medical Center, Tainan, Taiwan;

^16^Liverpool Clinical Laboratories, Liverpool University Hospitals Foundation Trust, Liverpool, UK;

^17^Department of Pathology and Laboratory Medicine, Indiana University, Indianapolis, IN, USA;

^18^Center for Genomic and Computational Biology and Department of Medicine, Duke University, Durham, NC, USA

^19^Amsterdam UMC, location Vrije Universiteit Amsterdam, Department of Pathology, Amsterdam, The Netherlands;

^20^Department of Clinical and Molecular Medicine, Sapienza University, School of Medicine and Psychology, Sant’ Andrea Hospital, Rome, Italy;

^21^Division of Cellular and Molecular Pathology, Department of Pathology, University of Cambridge, Cambridge, UK;

^22^Department of Pathology and Laboratory Medicine, University of Pennsylvania, Philadelphia, PA, USA;

^23^Department of Pathology, Massachusetts General Hospital and Harvard Medical School, Boston, MA, USA;

^24^Department of Pathology, Stanford University School of Medicine, Stanford, CA, USA;

^25^Department. of Dermatology, Northwestern University Feinberg Medical School, Chicago, IL, USA;

^26^Department of Pathology, Tata Memorial Hospital, Mumbai, India;

^27^Department of Pathology, Boston Children’s Hospital, Boston, MA, USA;

^28^Translational and Clinical Research Institute, Newcastle University Centre for Cancer, Faculty of Medical Sciences, Newcastle University, Newcastle-upon-Tyne, UK;

^29^Dr. Senckenberg Institute of Pathology, Goethe University Frankfurt, Frankfurt am Main, Germany;

^30^Hematology/Oncology, Universitätsklinikum Jena, Jena, Germany;

^31^Leiden University Medical Center, Department of Pathology, Leiden, The Netherlands;

^32^Department of Pathology and Laboratory Medicine, Nagoya, Japan;

^33^Kempf und Pfaltz Histologische Diagnostik Zurich, and Department of Dermatology, University Hospital Zurich, Zurich, Switzerland;

^34^Department of Hematopathology, Division of Pathology and Laboratory Medicine, The University of Texas MD Anderson Cancer Center, Houston, TX, USA;

^35^Department of Virology, Nagoya University Graduate School of Medicine, Nagoya, Japan;

^36^Department of Pathology, Hematopathology Section and Lymph Node Registry, University Hospital Schleswig-Holstein, University of Kiel, Kiel, Germany;

^37^Department of Pathology and Laboratory Medicine, Children’s Hospital Los Angeles, Los Angeles, CA, USA;

^38^Division of Hematology, Mayo Clinic, Rochester, MN, USA;

^39^Departments of Pathology & Genomic Medicine, The University of Texas MD Anderson Cancer Center, Houston, TX, USA;

^40^Department of Medical Biotechnology, University of Siena, Siena, Italy;

^41^Division of Nephrology and Hypertension, Division of Hematology, Mayo Clinic, Rochester, MN, USA;

^42^Department of Pathology, Medical College of Wisconsin and Children’s Wisconsin, Milwaukee, WI, USA;

^43^Department of Pathology, Fudan University Shanghai Cancer Center, Shanghai, PR China;

^44^Department of Pathology, West-China Hospital, Sichuan University, Chengdu, PR China;

^45^Department of Pathology & Immunology, Baylor College of Medicine and Texas Children’s Hospital, Houston, TX, USA;

^46^Department of Pathology, Charles University in Prague, Faculty of Medicine in Plzen, Plzen, Czech Republic;

^47^Department of Dermatology, Venereology and Allergology, University Medical Center Göttingen, Göttingen, Germany;

^48^Anatomic Pathology Department and Translational Hematopathology Lab, Valdecilla/IDIVAL University Hospital, Santander, Spain;

^49^Department of Laboratory Medicine and Pathology, Mayo Clinic, Rochester, MN, USA;

^50^Section of Pathology, Clinical Research Division, Fred Hutchinson Cancer Center, Seattle, WA, USA;

^51^Department of Pathology, Yong Loo Lin School of Medicine, National University of Singapore, Singapore, Singapore;

^52^Department of Clinical Pathology, Robert-Bosch-Krankenhaus, and Dr. Margarete Fischer-Bosch Institute of Clinical Pharmacology, Stuttgart, Germany;

^53^Department of Pathology, Bordeaux University Hospital, Bordeaux, France;

^54^Department of Pathology and Laboratory Medicine, Memorial Sloan Kettering Cancer Center, New York, NY, USA;

^55^Division of Hematology, Mayo Clinic, Rochester, Minnesota, Rochester, MN, USA;

^56^HMDS, Leeds Cancer Centre, Leeds Teaching Hospitals NHS Trust, Leeds, UK;

^57^Department of Pathology and Laboratory Medicine, University of California Los Angeles, Los Angeles, CA, USA;

^58^Institut Curie, Hematology department, Saint Cloud, France;

^59^Department of Pathology—Aga Khan University Hospital—Nairobi, Nairobi, Kenya;

^60^Section of Hematology/Oncology, University of Chicago, Chicago, IL, USA;

^61^Department of Oncology, University of Oxford, Oxford, UK;

^62^Immunology Division, Garvan Institute of Medical Research, Sydney, NSW, Australia;

^63^Institute of Human Genetics, Ulm University and Ulm University Medical Center, Ulm, Germany;

^64^Department of Hematology & Oncology, Shimane University School of Medicine, Shimane, Japan;

^65^Division of Haematology and Immunology, Leeds Institute of Medical Research, University of Leeds, Leeds, UK;

^66^Hospices Civils de Lyon/Department of Pathology/Université Lyon 1/Centre International de Recherche en Infectiologie (CIRI) INSERM U1111—CNRS UMR5308, Lyon, France;

^67^Department of Pathology, Hopital Haut-Lévêque, CHU Bordeaux, Pessac, France;

^68^National Amyloidosis Centre, University College London, London, UK;

^69^Department of Pathology, Institut Paoli-Calmettes and Aix-Marseille University, Marseille, France;

**AUTHOR CONTRIBUTIONS**

JK and AJL are standing members of the WHO Classification of Tumours editorial board. RA, JKCC, WJC, SEC, SSD, DDJ, MQD, JF, SG, AH, MSL, KNN, GO, SS, AS, WS, RSi and BW are expert members of the Haematolymphoid Tumours 5th edition blue book editorial board. CA, IA, ADA, IBOA, EB, GB, AMB, DB, MC, AC, WC, JKC, SSC, MC, AND, KSEJ, JG, DG, JG, MH, CJH, SH, PMJ, KK, WK, HK, WK, AEK, SK, SL, LL, NL, VL,XQL, WPL, ALj, AM, LJM, MM, RNM, CM, SMM, WM, VN, YN, SBN, IO, MP, MP, SVJ, ACR, KR, AR, JS, CS, CS, ARS, RSu, RT, ATG, FV, BV, ADW, LX and WX contributed as responsible authors in the book. All authors and editors contributed to discussions on the content of the book chapters. All listed authors edited and approved the manuscript.

The following colleagues are acknowledged for their expert contributions as authors in the WHO Classification of Haematolymphoid Tumours blue book on lymphoid topics, mesenchymal lesions specific to lymph node and spleen, and germline predisposition syndromes associated with the lymphoid neoplasms. Their affiliations were provided as given in the International Agency for Research on Cancer (IARC) databases. Where authors are identified as personnel of IARC/World Health Organization, the authors alone are responsible for the views expressed in this article, and they do not necessarily represent the decisions, policy, or views of the International Agency for Research on Cancer/World Health Organization.

Norah Olubunmi Akinola^70^, Yassmine Akkari^71^, Luis M. Allende^72^, Katsuyuki Aozasa^73^, Iguaracyra Araujo^74^, Luca Arcaini^75^, Kirit M. Ardeshna^76^, Naoko Asano^77^, Andishe Attarbaschi^78^, Chris M. Bacon^28^, Sharon Louise Barrans^56^, Tracy Batchelor^79^, Maxime Battistella^80^, Linda B. Baughn^49^, Amir Behdad^81^, Sigbjørn Berentsen^82^, Giada Bianchi^83^, Jacob Bledsoe^27^, Peter Borchmann^84^, Mark Bower^85^, Barbara Buldini^86^, Jan Andreas Burger^87^, Birgit Burkhardt^88^, Ryan D. Cassaday^89^, Giovanni Cazzaniga^90^, Nadine Cerf-Bensussan^91^, Ethel Cesarman^11^, Mammen Chandy^92^, Jennifer R. Chapman^93^, Björn Chapuy^94^, Xueyan Chen^95^, Chee Leong Cheng^96^, Carlos Chiattone^97^, Nicholas Chiorazzi^98^, Lucy B. Cook^99^, Wendy A. Cooper^100^, Gregory Philip Corboy^101^, Andrew John Cowan^89^, Immacolata Cozzolino^102^, Ian A Cree^103^, Emanuele S.G. d’Amore^104^, Andrew John Davies^105^, Martina Deckert^106^, Jan Delabie^107^, Elizabeth G. Demicco^107^, Vikram Deshpande^23^, Arjan Diepstra^108^, Daan Dierickx^109^, Kieron Dunleavy^110^, Barbara Eichhorst^111^, Daisuke Ennishi^112^, David C. Fajgenbaum^113^, Pedro Farinha^114^, Carlos Fernández de Larrea^115^, Kevin E. Fisher^45^, Jude Fitzgibbon^116^, Melina Flanagan^117^, Jonathan Fromm^95^, Juan F. Garcia^118^, William Robert Geddie^107^, Morie Gertz^38^, Ajay Gopal^89^, Satish Gopal^119^, Patricia Theresa Greipp^49^, Alejandro Gru^120^, Ritu Gupta^121^, Martin-Leo Hansmann^122^, Konnie M. Hebeda^123^, Klaus Herfarth^124^, Marco Herling^125^, Olivier Hermine^126^, Khe Hoang-Xuan^127^, Jennelle Hodge^128^, Shimin Hu^34^, Yuhua Huang^129^, Yin Pun Hung^23^, Stephen Hunger^130^, Hiroto Inaba^131^, Hiroshi Inagaki^132^, Javeed Iqbal^133^, Kenji Ishitsuka^134^, Noriko Iwaki^135^, Keiji Iwatsuki^136^, Nitin Jain^87^, Yoon Kyung Jeon^137^, Marshall Kadin^138^, Sachiko Kaji^139^, Aanchal Kakkar^121^, Anastasios Karadimitris^140^, Keisuke Kataoka^141^, Seiichi Kato^142^, Marie José Kersten^143^, Rhett P. Ketterling^49^, Ji Eun Kim^144^, Christian P. Kratz^145^, Robert Kridel^146^, Sigurdur Kristinsson^147^, Ralf Küppers^148^, Isinsu Kuzu^149^, Yok-Lam Kwong^150^, Ann Lacasce^151^, Laurence Lamant-Rochaix^152^, Thierry Lamy^153^, Ola Landgren^154^, Siddhartha Laskar^155^, William Bradlyn Laskin^156^, Georg Lenz^157^, Shaoying Li^34^, Gan Di Li^44^, Pei Lin^34^, Franco Locatelli^158^, Robert Brian Lorsbach^159^, Izidore Lossos^154^, Thomas P. Jr Loughran^160^, William R. Macon^49^, Joseph J. Maleszewski^49^, Pankaj Malhotra^161^, Teresa Marafioti^162^, Dai Maruyama^163^, Alexander Marx^164^, Sam M. Mbulaiteye^165^, Veronique Meignin^80^, Ester Mejstrikova^166^, Pamela Michelow^167^, Markku Miettinen^168^, Rodney R. Miles^169^, Hiroaki Miyoshi^170^, Thierry Jo Molina^171^, Manuela Mollejo^172^, Shuji Momose^173^, Tetsuya Mori^174^, William G. Morice^49^, Bertrand Nadel^175^, Hirokazu Nagai^176^, Motoo Nagane^177^, Reena Nair^92^, Naoya Nakamura^178^, Atsuko Nakazawa^179^, Samih Nasr^49^, Andrew Gordon Nicholson^180^, Alina Nicolae^181^, Robert Shigeo Ohgami^182^, Naoki Oishi^183^, Timothy S. Olson^130^, Nicolas Ortonne^184^, Bruno Paiva^185^, Qiang Pan-Hammarström^186^, Mayur Parihar^187^, Marco Paulli^188^, Andrea Petersen^189^, Jennifer Picarsic^190^, Alessandro Pileri^191^, Nicola Pimpinelli^192^, Jose A Plaza^193^, Karen R. Rabin^194^, Markus Raderer^195^, Kanti Rai^196^, Ulla Randen^197^, Huilan Rao^129^, Alistair Robson^198^, Rosemary Rochford^199^, Richard Rosenquist^200^, Davide Rossi^201^, Esther D. Rossi^202^, Simon Rule^203^, Grzegorz Rymkiewicz^204^, Elena Sabattini^205^, Vaskar Saha^206^, Mamiko Sakata-Yanagimoto^207^, Christian A. Sander^208^, J. Martin Sangueza^209^, Omar P. Sangüeza^210^, Marco Santucci^211^, Yasuharu Sato^212^, Akira Satou^142^, Kristian Theo Schafernak^213^, Fernando Schmitt^214^, Gianpietro Semenzato^215^, Manju Sengar^92^, Tait Shanafelt^216^, Kazuyuki Shimada^217^, Graham W. Slack^114^, Susan Slager^218^, Riccardo Soffietti^219^, David A. Solomon^182^, Kostas Stamatopoulos^220^, Christian Steidl^221^, Stephan Stilgenbauer^222^, Narittee Sukswai^223^, Kengo Takeuchi^224^, Giovanni Tallini^225^, Junichi Tamaru^226^, Soo-Yong Tan^51^, Prashant Tembhare^227^, Enrico Tiacci^228^, Yoshiki Tokura^229^, Olivier Tournilhac^230^, Steven Treon^231^, Lorenz Truemper^232^, Kunihiro Tsukasaki^233^, Frits van Rhee^234^, Abraham Varghese^235^, Maarten H. Vermeer^236^, Philippe Vielh^237^, Brian Walker^238^, Michael Wang^239^, Huan-You Wang^240^, Zhe Wang^241^, Takashi Watanabe^242^, Oliver Weigert^243^, David Weinstock^244^, Sean J. Whittaker^245^, Rein Willemze^236^, Wilhelm Woessmann^246^, Catherine J. Wu^244^, Motoko Yamaguchi^247^, Hidetaka Yamamoto^248^, Daisuke Yamashita^249^, Shenmiao Yang^250^, David T Yang^251^, Takahiko Yasuda^252^, Wei-Hua Yin^253^, Yoh Zen^254^, Sha Zhao^44^, Wei-Li Zhao^255^

^70^Haematology and Immunology, Obafemi Awolowo University, Ile Ife, Nigeria;

^71^Cytogenetics and Molecular Pathology, Legacy Health, Portland, OR, USA;

^72^Immunology Department, Hospital 12 de Octubre, imas12, Madrid, Spain;

^73^Department of Pathology, Osaka University Graduate School of Medicine, Suita, Osaka, Japan;

^74^Pathology and Forensic Medicine, Federal University of Bahia, Salvador, Bahia, Brazil;

^75^Fondazione IRCCS Policlinico San Matteo and Departement of Molecular Medicine, University of Pavia, University of Pavia, Pavia, Italy;

^76^Cancer Division, University College London Hospitals, London, UK;

^77^Molecular Diagnostics, Nagano Prefectural Shinshu Medical Center, Suzaka, Japan;

^78^Pediatric Hematology and Oncology, St. Anna Children’s Hospital, Vienna, Austria;

^79^Department of Neurology, Brigham and Women’s Hospital, Boston MA, USA;

^80^Pathology Department, Hôpital Saint Louis, AP-HP Université Paris, Paris, France;

^81^Department of Pathology, Northwestern University, Chicago, IL, USA;

^82^Department of Research and Innovation, Helse Fonna Hospital Trust, Haugesund, Norway;

^83^Division of Hematology, Department of Medicine, Brigham and Women’s Hospital, Boston MA, USA;

^84^1st Dept of Internal Medicine, University Hospital of Cologne, Cologne, Germany;

^85^National Centre for HIV Malignancy, Chelsea & Westminster Hospital, London, UK;

^86^Maternal and Child’s Health Department, University of Padova, Padova, Italy;

^87^Department of Leukemia, The University of Texas MD Anderson Cancer Center, Houston, TX, USA;

^88^Pediatric Hematology, Oncology and BMT, University Hospital Muenster, Muenster, Germany;

^89^Department of Medicine, University of Washington School of Medicine, Seattle, WA, USA;

^90^Pediatrics and Medical Genetics, Univ Milan Bicocca—Centro Ricerca Tettamanti, Barzano, Italy;

^91^Laboratory Intestinal Immunity, Institut Imagine-Inserm U1183, Université de Paris, Paris, France;

^92^Clinical Hematology & Medical Oncology, Tata Medical Center, Kolkata, India;

^93^Pathology and Laboratory Medicine, University of Miami, Miami, FL, USA;

^94^Hematology, Oncology and Tumor Immunology, Charité, University Medical Center Berlin, Berlin, Germany;

^95^Laboratory Medicine and Pathology, University of Washington, Seattle, WA, USA;

^96^Anatomical Pathology, Singapore General Hospital, Singapore, Singapore;

^97^Hematology and Oncology, Santa Casa Midical School, Sao Paulo, Brazil;

^98^Karches Center for Oncology Research, The Feinstein Institutes for Medical Research, Manhasset, NY, USA;

^99^Department of Haematology, Imperial College Healthcare NHS Trust, London, UK;

^100^Tissue Pathology and Diagnostic Oncology, Royal Prince Alfred Hospital, NSW Health Pathology, Camperdown, NSW, Australia;

^101^Department of Haematology, Pathology Queensland, Herston, QLD, Australia;

^102^Pathology Unit, Department of Mental and Physical Health and Preventive Medicine, University of Campania “L. Vanvitelli”, Naples, Italy

^103^Evidence Synthesis and Classification Branch, International Agency for Research on Cancer, Lyon, France;

^104^UOC di Anatomia Patologica, Ospedale San Bortolo, Vicenza, Italy;

^105^Cancer Sciences Unit, University of Southampton, Southampton, UK;

^106^Department of Neuropathology, University Hospital of Cologne, Cologne, Germany;

^107^Laboratory Medicine Program, University Health Network and University of Toronto, Toronto, ON, Canada;

^108^Pathology and Laboratory Medicine, University Medical Center Groningen, Groningen, The Netherlands;

^109^Department of Hematology, University Hospitals Leuven, Leuven, Belgium;

^110^Hematology and Oncology, Lombardi Cancer Center, Georgetown University, Washington, DC, USA;

^111^Department I for Internal Medicine and Center for Integrated Oncology Aachen, Bonn, Cologne, Duesseldorf, University of Cologne, Cologne, Germany;

^112^Department of Hematology and Oncology, Okayama University Hospital, Okayama, Japan;

^113^Department of Medicine, University of Pennsylvania, Philadelphia, PA, USA;

^114^Department of Pathology, BC Cancer Agency, Vancouver, BC, Canada;

^115^Amyloidosis and Myeloma Unit, Department of Hematology, Hospital Clínic of Barcelona, IDIBAPS, University of Barcelona, Barcelona, Spain;

^116^Genomics and Computational Biology, Queen Mary University of London, London, UK;

^117^Department of Pathology, West Virginia University School of Medicine, Morgantown, WV, USA;

^118^Department of Pathology, MD Anderson Cancer Center Madrid, Madrid, Spain;

^119^Center for Global Health, National Cancer Institute, Rockville, MD, USA;

^120^Pathology & Dermatology, University of Virginia, Charlottesville, VA, USA;

^121^Laboratory Oncology, All India Institute of Medical Sciences, Delhi, New Delhi, India;

^122^Consultation Center for Hematopathology, Institute of Pathology and Molecular Pathology, Wuppertal, Germany;

^123^Department of Pathology, The Radboud University Medical Center, Nijmegen, The Netherlands;

^124^Radiation Oncology, University Hospital Heidelberg, Heidelberg, Germany;

^125^Hematology, Cellular Therapy, Hemostaseology, University of Leipzig, Leipzig, Germany;

^126^Hematology and Laboratory of Physiopathology and Treatment of Hematological Disorders, Necker Hospital, APHP, INSERM U1163, Imagine, Paris University, Paris, France;

^127^Neuro-oncology, Hôpital Universitaire Pitié Salpêtrière, Paris, France;

^128^Department of Medical and Molecular Genetics, Indiana University School of Medicine, Indianapolis, IN, USA;

^129^Department of Pathology, Sun Yat-sen University Cancer Center, Guangzhou, PR China;

^130^Division of Oncology, Department of Pediatrics, Children’s Hospital of Philadelphia, Philadelphia, PA, USA;

^131^Department of Oncology, St. Jude Children’s Research Hospital, Memphis, TN, USA;

^132^Department of Pathology and Molecular Diagnostics, Nagoya City University, Nagoya, Japan;

^133^Pathology and Microbiology, University of Nebraska Medical Center, Omaha, NE, USA;

^134^Department of Hematology and Rheumatology, Kagoshima University, Kagoshima, Japan;

^135^Department of Hematology, National Cancer Center Hospital, Tsukiji, Chuo-ku, Tokyo, Japan;

^136^Departments of Dermatology, Okayama University Graduate School of Medicine, Dentistry and Pharmaceutical Sciences, Okayama, Japan;

^137^Department of Pathology, Seoul National University College of Medicine, Seoul, South Korea;

^138^Department of Pathology and Laboratory Medicine, Brown University Alpert School of Medicine, Providence, RI, USA;

^139^Department of Pathology, Japanese Red Cross Narita Hospital, Narita, Chiba, Japan;

^140^Centre for Haematology, Department of Immunology and Inflammation, Imperial College London, London, UK;

^141^Division of Hematology, Department of Medicine, Keio University School of Medicine, Tokyo, Japan;

^142^Department of Pathology and Molecular Diagnostics, Aichi Cancer Center Hospital, Nagoya, Japan;

^143^Department of Hematology, Amsterdam University Medical Centers, Amsterdam, The Netherlands;

^144^Department of Pathology, Seoul National University SNU SMG Boramae Hospital, Seoul, South Korea;

^145^Department of Pediatric Hematology and Oncology, Hannover Medical School, Hannover, Germany;

^146^Division of Medical Oncology and Hematology, Princess Margaret Cancer Centre—UHN, Toronto, ON, Canada;

^147^Faculty of Medicine, University of Iceland, Reykjavik, Iceland;

^148^Institute of Cell Biology (Cancer Research), University of Duisburg-Essen, Essen, Germany;

^149^Department of Pathology, University of Ankara, School of Medicine, Ankara, Turkey;

^150^Department of Medicine, University of Hong Kong, Queen Mary Hospital, Hong Kong, Hong Kong;

^151^Medical Oncology, Dana Farber Cancer Institute, Boston, MA, USA;

^152^Departement of Pathology, Institut Universitaire du cancer Toulouse Oncopole, Toulouse, France;

^153^Department of Hematology, Rennes University Hospital, Rennes, France;

^154^Department of Medicine, Sylvester Comprehensive Cancer Center, University of Miami, Miami, FL, USA;

^155^Radiation Oncology, Tata Memorial Centre, Mumbai, India;

^156^Department of Pathology, Yale School of Medicine, New Haven, CT, USA;

^157^Department of Hematology, Oncology and Pneumology, University Hospital Münster, Münster, Germany;

^158^Pediatric Hematology/Oncology, Cell and Gene Therapy, Bambino Gesù Children’s Hospital, Rome, Italy;

^159^Division of Pathology, Cincinnati Children’s Hospital Medical Center, University of Cincinnati College of Medicine, Cincinnati, OH, USA;

^160^Hematology/Oncology, University of Virginia, Charlottesville, VA, USA;

^161^Clinical Hematology and Medical Oncology, Postgraduate Institute of Medical Education and Research, Chandigarh, India;

^162^Department of Cellular Pathology, University College Hospital London, London, UK;

^163^Hematology Oncology, Cancer Institute Hospital of Japanese Foundation of Cancer Research, Tokyo, Japan;

^164^Department of Pathology, University Medical Centre Mannheim, Mannheim, Germany;

^165^Infections and Immunoepidemiology Branch, DCEG, National Cancer Institute, Rockville, MD, USA;

^166^Department of Pediatric Hematology and Oncology, 2nd Faculty of Medicine, University Hospital Motol, Prague, The Czech Republic;

^167^Department of Anatomical Pathology, University of the Witwatersrand and National Health Laboratory Service, Johannesburg, South Africa;

^168^Laboratory of Pathology, National Cancer Institute/NIH, Bethesda, MD, USA;

^169^Department of Pathology, University of Utah, Salt Lake City, UT, USA;

^170^Department of Pathology, Kurume University, School of Medicine, Kurume, Japan;

^171^Department of Pathology, AP-HP, Hôpital Necker-Enfants Malades and Robert Debré, Université de Paris, Paris, France;

^172^Department of Pathology, Hospital Universitario de Toledo, Toledo, Spain;

^173^Department of Pathology, Saitama Medical University, Saitama Medical Center, Kawagoe, Japan;

^174^Department of Pediatrics, St. Marianna University School of Medicine, Kawasaki, Kanagawa, Japan;

^175^Centre d’Immunologie de Marseille-Luminy, Inserm CNRS AMU, Marseille, France;

^176^Clinical Research Center, National Hospital Organization Nagoya Medical Center, Nagoya, Japan;

^177^Department of Neurosurgery, Kyorin University Faculty of Medicine, Tokyo, Japan;

^178^Department of Pathology, Tokai University School of Medicine, Isehara, Japan;

^179^Clinical Research, Saitama Children’s Medical Center, Saitama, Japan;

^180^Department of Histopathology, Royal Brompton & Harefield NHS Foundation Trust, London, UK;

^181^Department of Pathology, Hautepierre, University Hospital Strasbourg, Strasbourg, France;

^182^Department of Pathology, University of California San Francisco, San Francisco, CA, USA;

^183^Department of Pathology, University of Yamanashi, Chuo, Yamanashi, Japan;

^184^Department of Pathology, AP-HP, Hopitaux Universitaires Henri-Mondor, Creteil, France;

^185^Hemato-Oncology, Clinica Universidad de Navarra, Pamplona, Spain;

^186^Department of Biosciences and Nutrition, Karolinska Institutet, Huddinge, Sweden;

^187^Lab Haematology/Cytogenetics & Molecular Genetics, Tata Medical Center, Kolkata, India;

^188^Pathology Unit, Department of Molecular Medicine, University of Pavia, Pavia, Italy;

^189^Pediatric Development and Rehab, Genetics Division, Legacy Health/Randall Children’s Hospital and Washington State University Elson S. Floyd College of Medicine, Portland, OR, USA;

^190^Department of Pathology, Cincinnati Childrens Hospital Medical Center, Cincinnati, OH, USA;

^191^Experimental, Diagnostic and Specialty Medicine Department, Dermatology Unit, Bologna, Italy;

^192^Health Sciences, University of Florence Medical School, Florence, Italy;

^193^Pathology and Dermatology, The Ohio State University Medical Center, Columbus, OH, USA;

^194^Baylor College of Medicine, Texas Children’s Cancer Center, Houston, TX, USA;

^195^Internal Medicine I, Division of Oncology, Medical University of Vienna, Vienna, Austria;

^196^Department of Medicine, Northwell Health Cancer Institute, New Hyde Park, NY, USA;

^197^Department of Pathology, Akershus University Hospital/University of Oslo, Lørenskog, Norway;

^198^Departamento de Diagnostico Laboratorial, IPOLFG—Servico de Anatomia Patologica, Lisboa, Portugal;

^199^Immunology and Microbiology, University of Colorado, Anschutz Medical Campus, Aurora, CO, USA;

^200^Department of Molecular Medicine and Surgery, Karolinska Institutet, Stockholm, Sweden;

^201^Department of Hematology, Oncology Institute of Southern Switzerland, Bellinzona, Switzerland;

^202^Department of Anatomic Pathology and Histology, Fondazione Policlinico Universitario Agostino Gemelli-IRCCS, Rome, Italy;

^203^Department of Haematology, Plymouth University Medical School, Plymouth, UK;

^204^Department of Pathology and Laboratory Diagnostics, Maria Sklodowska-Curie National Research Institute of Oncology, Warsaw, Poland;

^205^Haematopathology, IRCCS Azienda Ospedaliero-Universitaria, Bologna, Italy;

^206^Paediatric Haematology and Oncology, Tata Translational Cancer Research Centre, Kolkata, India;

^207^Department of Hematology, University of Tsukuba, Ibaraki, Japan;

^208^Department of Dermatology, Asklepios Klinik St. Georg, Hamburg, Germany;

^209^Pathology and Dermatology, Hospital Obrero Nro 1 CNS, Hospital General, La Paz, Spain;

^210^Dept of Pathology and Dermatology, Wake Forest University, School of Medicine Medical Center Boulevard, Winston-Salem, NC, USA;

^211^Department of Health Sciences, Division of Histopathology and Molecular Diagnostics, University of Florence School of Human Health Sciences, Firenze, Italy;

^212^Department of Pathology, Okayama University Graduate School of Health Sciences, Okayama, Japan;

^213^Pathology and Laboratory Medicine, Phoenix Children’s Hospital, Phoenix, AZ, USA;

^214^Molecular Pathology Unit, Medical Faculty, Porto University, Porto, Portugal;

^215^Department of Hematology, Veneto Institute of Molecular Medicine, University of Padua, Padova, Italy;

^216^Department of Medicine, Division of Hematology, Stanford University, Palo Alto, CA, USA;

^217^Department of Hematology and Oncology, Nagoya University Graduate School of Medicine, Nagoya, Japan;

^218^Medicine and Quality Health Sciences, Mayo Clinic, Rochester, MN, USA;

^219^Department of Neuro-Oncology, University of Turin and City of Health and Science Hospital, Turin, Italy;

^220^Centre for Research and Technology Hellas, Institute of Applied Biosciences, Thessaloniki, Greece;

^221^Pathology and Laboratory Medicine, University of British Columbia, Vancouver, BC, Canada;

^222^Comprehensive Cancer Center Ulm (CCCU), Ulm University, Ulm, Germany;

^223^Department of Pathology, Chulalongkorn University, Bangkok, Thailand;

^224^Division of Pathology, Cancer Institute, Japanese Foundation for Cancer Research, Tokyo, Japan;

^225^Department of Pathology, Policlinico S.Orsola-Malpighi, Bologna, Italy;

^226^Pathology, Biomedical and Sciences, Saitama Medical Center, Saitama Medical University, Kawagoe, Japan;

^227^Hematopathology Laboratory, Advanced Centre for Treatment, Research and Education in Cancer, Tata Memorial Centre, Navi Mumbai, India;

^228^Department of Medicine and Surgery, Institute of Hematology and Center for Hemato-Oncology Research, Perugia, Italy;

^229^Allergic Disease Research Center, Chutoen General Medical Center, Kakegawa, Japan;

^230^Hematology and Cell Therapy, CHU de Clermont-Ferrand, Clermont-Ferrand, France;

^231^Department of Medicine, Harvard Medical School, Boston, MA, USA;

^232^Hematology and Medical Oncology, Universitätsmedizin Göttingen, Göttingen, Germany;

^233^Department of Hematology, International Medical Center, Saitama Medical University, Hidaka, Japan;

^234^Myeloma Center, UAMS Winthrop P. Rockefeller Cancer Institute, Little Rock, AR, USA;

^235^Department of Haematology, Little Flower Hospital and Research Centre, Angamaly, India;

^236^Department of Dermatology, Leiden University Medical Center, Leiden, The Netherlands;

^237^Department of Pathology, Medipath and American Hospital of Paris, Paris, France;

^238^Division of Hematology Oncology, Indiana University, Indianapolis, IN, USA;

^239^Lymphoma and Myeloma, University of Texas MD Anderson Cancer Center, Houston, TX, USA;

^240^Department of Pathology, University of California San Diego, San Diego, CA, USA;

^241^Department of Pathology, Fourth Military Medical University, Xi’an, PR China;

^242^Personalized Cancer Immunotherapy, Mie University Graduate School of Medicine, Tsu, Japan;

^243^Medical Department III, Ludwig-Maximilians-University (LMU) Hospital, Munich, Germany;

^244^Medical Oncology, Dana-Farber Cancer Institute and Havard Medical School, Boston, MA, USA;

^245^School of Basic and Medical Biosciences KCL, St. John’s Institute of Dermatology, London, UK;

^246^Pediatric Hematology and Oncology, University Medical Center Hamburg-Eppendorf, Hamburg, Germany;

^247^Department of Hematological Malignancies, Mie University Graduate School of Medicine, Tsu, Japan;

^248^Anatomic Pathology, Kyushu University, Fukuoka, Japan;

^249^Department of Pathology, Kobe City Medical Center General Hospital, Kobe, Japan;

^250^Department of Hematology, Peking University Peoples’ Hospital, Peking Institute of Hematology, Beijing, PR China;

^251^Department of Pathology and Laboratory Medicine, University of Wisconsin, Madison, WI, USA;

^252^Advanced Diagnosis, Nagoya Medical Center, Nagoya, Japan;

^253^Department of Pathology, Peking University Shenzhen Hospital, Shenzhen, PR China;

^254^Institute of Liver Studies, King’s College Hospital, London, UK;

^255^National Center of Translational Medicine, Institute of Hematology, Shanghai, PR China.

